# Resmetirom Ameliorates Fibrogenesis in Hepatic Stellate Cells via Thyroid Hormone Receptor Alpha‐Fatty‐Acid Amide Hydrolase 1 Signaling Pathway

**DOI:** 10.1002/mco2.70901

**Published:** 2026-08-05

**Authors:** Eva Novoa, Alba Cabaleiro, Borja López‐Picallo, Natalia da Silva Lima, Cristina Riobello, Carmen Pena, Jose Iglesias‐Moure, Ana Senra, Maria Luz Martínez‐Chantar, Anabel Fernandez‐Iglesias, Jordi Gracia‐Sancho, Susana Bravo, Carlos Dieguez, Marta Varela‐Rey, Vincent Prevot, Markus Schwaninger, Miguel López, Rubén Nogueiras

**Affiliations:** ^1^ Department of Physiology, CIMUS University of Santiago de Compostela Santiago de Compostela Spain; ^2^ CIBER Fisiopatología de la Obesidad y Nutrición (CIBERobn) Madrid Spain; ^3^ Regulation of Gene Expression and Applications (EXPRELA) Group, Interdisciplinary Centre for Chemistry and Biology – CICA, University of Coruña A Coruña Spain; ^4^ Department of Biochemistry and Molecular Biology CIMUS, IDIS, Universidade de Santiago de Compostela Santiago de Compostela Spain; ^5^ Proteomic Unit, Health Research Institute of Santiago de Compostela (IDIS), Santiago de Compostela A Coruña Spain; ^6^ Liver Disease Lab, BRTA CIC bioGUNE, Centro de Investigación Biomédica en Red de Enfermedades Hepáticas y Digestivas (CIBERehd) Derio Spain; ^7^ Liver Research Unit, Institut de Recerca Biomèdica Catalunya Sud (IRBCatSud), Hospital Universitari Joan 23 de Tarragona ‐ CIBEREHD Tarragona Spain; ^8^ Laboratory of Development and Plasticity of the Neuroendocrine Brain, Univ. Lille, Inserm, CHU Lille, Lille Neuroscience & Cognition UMR‐S 1172, European Genomic Institute for Diabetes (EGID) Lille France; ^9^ Institute for Experimental and Clinical Pharmacology and Toxicology, University of Lübeck Lübeck Germany; ^10^ Galician Agency of Innovation (GAIN), Xunta de Galicia Santiago de Compostela Spain

**Keywords:** hepatic stellate cells, liver fibrosis, resmetirom, thyroid hormones

## Abstract

Resmetirom is a liver‐directed, thyroid hormone receptor β (THRβ)‐selective agonist approved for treating metabolic‐associated steatohepatitis (MASH). While Resmetirom hepatocyte‐specific effects are well‐established, its impact on other hepatic cells, particularly hepatic stellate cells (HSCs), the main fibrogenic cells, remains unknown.

Using animal models, immortalized cell lines, and primary murine and human HSCs, we combined pharmacological treatments with genetic manipulation of THRα and fatty acid amide hydrolase (FAAH) to examine the effects of Resmetirom. Resmetirom ameliorates MASH through actions in both hepatocytes and HSCs. The overexpression of solute carrier organic anion transporter family member 1B1(SLCO1β1) in both cell types increases the efficiency of Resmetirom. THRα expression is higher in HSCs than in hepatocytes in both human and murine cells. Resmetirom attenuates TGF‐β1‐induced HSC activation via THRα and increased FAAH expression and activity, while their inhibition prevents Resmetirom from reducing fibrotic marker expression and the elevated glycolytic activity typical of activated HSCs.

These results uncover an unrecognized mechanism of action for Resmetirom, demonstrating that its antifibrotic efficacy extends beyond hepatocytes to include direct effects on HSCs via THRα and FAAH. These results may support the development of future therapeutic strategies aimed at THRα in HSC, in combination with existing approaches targeting hepatocyte THRβ.

## Graphical abstract

1. Resmetirom attenuates the activation of HSCs induced in in vitro and in vivo models.

2. Resmetirom attenuates the activation of HSCs through its specific action on the thyroid hormone receptor alpha.

3. Resmetirom requires fatty‐acid amide hydrolase 1 to reduce HSC activation.

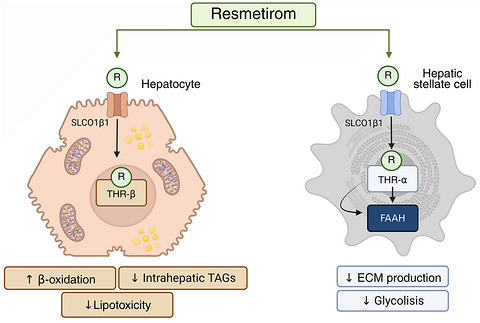

## 1 Introduction

Metabolic‐dysfunction‐associated steatotic liver disease (MASLD) has become the most common chronic liver condition globally, affecting approximately 25% of the global population, with even higher prevalence observed in specific regions and demographic groups [1]. MASLD encompasses a broad spectrum of liver disorders, among which metabolic‐associated steatohepatitis (MASH) represents its inflammatory subtype, characterized by hepatic inflammation and accelerated fibrosis progression [2]. The etiopathogenesis of MASH is multifaceted and intricate, involving numerous factors. Notably, strong associations have been identified between MASH and metabolic disorders such as obesity, type 2 diabetes mellitus, and dyslipidemia [3, 4, 5].

Thyroid hormones (THs) regulate essential biological functions, particularly in the regulation of hepatic glucose and lipid metabolism, as well as in the maintenance of energy homeostasis [6, 7]. These hormones exert their actions through TH receptors (THRs), which belong to the nuclear receptor superfamily and function as ligand‐dependent transcription factors [8]. Two main isoforms of THRs, THRα and THRβ, are distributed throughout the body, with each isoform predominating in different tissues. Notably, THRβ is highly expressed in hepatocytes, where it plays a critical role in regulating metabolic pathways that are frequently impaired in the liver in MASLD/MASH [9].

Resmetirom, initially identified as MGL‐3196, represents a notable advancement in MASH treatment. This orally administered, liver‐targeted, THRβ‐selective agonist received accelerated approval from the United States Food and Drug Administration (FDA) in 2024 and the European Medicines Agency (EMA) in 2025 for the treatment of MASH with moderate to advanced liver fibrosis [10, 11]. The approval was granted based on the results of the MAESTRO‐Non‐Alcoholic Steatohepatitis (NASH) clinical trial [12] supplemented by safety data from the MAESTRO‐Non‐Alcoholic Fatty Liver Disease (NAFLD)‐1 trial [13]. In the MAESTRO‐NASH trial, the resolution of NASH without worsening fibrosis was observed in 25.9% of the patients receiving 80 mg Resmetirom group and in 29.9% of those receiving 100 mg, compared with 9.7% of those in the placebo group. Additionally, fibrosis improvement by at least one stage, without exacerbation of the NAFLD activity score, was achieved in 24.2% of patients in the 80 mg Resmetirom group and in 25.9% of those in the 100 mg group [12].

Although Resmetirom has demonstrated higher selectivity for THRβ over THRα in an in vitro functional coactivator recruitment assay [14], it also exhibits a lower binding affinity for THRα. Notably, hepatic stellate cells (HSCs) predominantly express THRα [15]. Upon liver injury, HSCs undergo activation, transitioning into myofibroblast‐like cells that proliferate and secrete excessive amounts of extracellular matrix proteins, particularly collagen, contributing to the accumulation of scar tissue characteristic of liver fibrosis [16, 17, 18]. Consequently, this study aims to evaluate the hypothesis that the antifibrotic effects of Resmetirom may be, at least partially, directly mediated through its actions on HSCs.

## 2 Results

### 2.1 Expression of THRβ and THRα in the Liver of People With MASLD/MASH

To elucidate the expression of *THRα* and *THRβ* in specific hepatic cell populations, we analyzed a recently published single‐cell RNA‐seq dataset comprising liver samples from both healthy individuals and those with cirrhotic‐MASLD (GEO accessions: GSE185477 and GSE174748) [19, 20]. The results indicate that *THRβ* is expressed at a significantly higher proportion overall, with the highest expression observed in hepatocytes (92.8%) and cholangiocytes (68.6%). HSCs also exhibit notable expression of this gene proportion (54.8%) (Figure S1A,B). In contrast, *THRα* expression is more restricted, with cholangiocytes (9.2%) and HSC (5.8%) displaying the highest relative levels (Figure S1C,D).

### 2.2 Resmetirom Reduces Lipid Content in Human Hepatocytes by Its Action on *THRβ*

Given that both *THRα* and *THRβ* are expressed in hepatocytes and HSCs, we sought to determine whether Resmetirom exerts direct effects on these cell types. To establish the functional concentrations of Resmetirom, we treated THLE‐2 cells, a human hepatocyte cell line, with either a combination of oleic acid (OA) and palmitic acid (PA) to induce lipid accumulation. Cells were then co‐treated with Resmetirom at concentrations of 50 and 100 µM. As anticipated, the lipid content triggered by the fatty acid mixture was partially reduced by Resmetirom at 100 µM (Figure S2A).

To further investigate the molecular mechanisms underlying the effects of Resmetirom, we analyzed the expression of thyroid hormone receptor‐binding protein (*THRBP*), a versatile coactivator participating in the transcriptional activity of multiple promoters, and we also measured the expression of DIO1, the hepatic deiodinase that modulates local thyroid hormone availability [21]. Resmetirom treatment led to a significant upregulation of *THRBP* expression and DIO1 protein levels (Figure S2B,C). Importantly, these effects were not associated with alterations in cell viability, as determined by crystal violet and MTT assays (Figure S2D,E), nor with apoptosis, as evidenced by unchanged protein levels of cleaved caspase‐3 and cleaved caspase‐7 between vehicle‐ and Resmetirom‐treated groups (Figure S2F).

A steatosis mechanism highly relevant to MASLD is de novo lipogenesis, and MASLD is commonly associated with hyperglycemia and hyperinsulinemia. Therefore, we modeled this situation by conducting an experiment using THLE‐2 cells under these conditions and measured the enzymes involved in lipid metabolism. We then incubated hepatocytes with high glucose/high insulin and treated them with Resmetirom. As expected, in a medium with high glucose/high insulin, lipid content was augmented; however, Resmetirom significantly reduced lipid content, as measured by oil red O staining (Figure S2G). We also performed this experimental approach in primary human hepatocytes, and similarly, the high‐fat content observed under hyperglycemia and hyperinsulinemia was reversed when Resmetirom was added to cells (Figure S2H).

Next, we sought to examine the role of *THRα* and *THRβ* in regulating the effects of Resmetirom on fatty acid metabolism in hepatocytes. To achieve this, we selectively silenced *THRα, THRβ*, or both receptors simultaneously in THLE‐2 cells (Figure S3A–C) treated with OA, PA, and Resmetirom. The fatty acid combination elicited the anticipated increase in lipid content, which was subsequently attenuated by Resmetirom treatment. However, the effects of Resmetirom differed based on the selective genetic inhibition of THRs. Specifically, silencing *THRα* in hepatocytes did not prevent Resmetirom from reducing lipid content (Figure S3A). In contrast, silencing *THRβ* either alone or in combination with *THRα* abolished the ability of Resmetirom to reduce lipid accumulation (Figure S3B,C). These findings are consistent with previous reports [14, 22] suggesting that Resmetirom requires intact *THRβ* signaling to exert its protective effects in hepatocytes [23, 24].

### 2.3 Resmetirom Ameliorates Liver Fibrosis in Mice Through Its Actions Within Hepatocytes and HSCs

The effect of Resmetirom was initially assessed in a mouse model of diet‐induced MASLD and MASH. Mice were fed a Western diet (WD) (Table S1) for 5 weeks and subsequently treated with either a vehicle or a dose‐response of Resmetirom using 0.1, 0.5, 1, and 3 mg/kg for an additional 3 weeks (Figure S4A). As expected, mice fed with a Western diet showed increases in body weight; however, treatment with Resmetirom at the tested doses did not lead to significant changes in these parameters (Figure S4B). While liver weight, serum cholesterol, and triglyceride levels were significantly reduced at doses of 0.5, 1, and 3 mg/kg (Figure S4C–E), circulating alanine transaminase (ALT) and aspartate transaminase (AST) levels were significantly decreased only at the highest dose (Figure S4F). Consistently, lipid deposition was reduced at all three doses of Resmetirom, as shown by Oil Red O staining, whereas collagen content in liver sections was affected only by the highest dose, as demonstrated by Sirius Red staining (Figure S4G).

**FIGURE 1:**
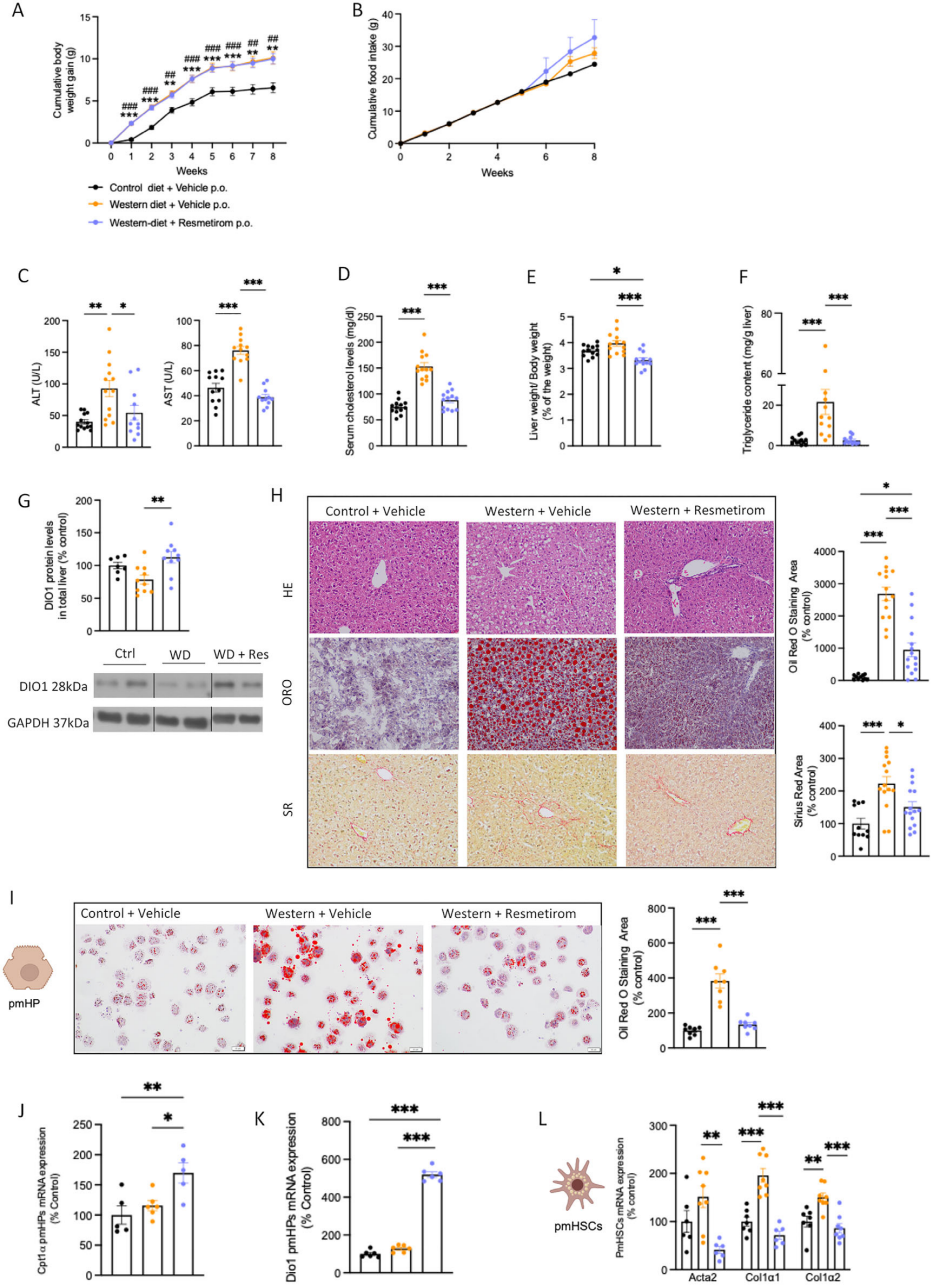
Resmetirom improves steatosis and fibrosis in mice fed a Western diet for 8 weeks. Mice were fed a Chow diet (CD) or a Western diet (WD) for 5 weeks to induce MASLD/MASH, followed by treatment with either a vehicle or Resmetirom for 3 weeks (*n* = 11–16 per group). (A, B) Cumulative body weight gain and cumulative food intake in WD‐fed mice with or without Resmetirom treatment. (C) Serum levels of ALT and AST as indicators of liver injury. (D) Circulating cholesterol levels. (E) Liver weight. (F) Hepatic triglyceride content levels. (G) Hepatic DIO1 protein levels. (H) Representative micrographs of Oil Red O staining (lipid accumulation) and Sirius Red staining (collagen deposition) in liver sections. (I) Oil Red O staining in primary hepatocytes (pmHP) isolated from WD‐fed mice, with or without Resmetirom treatment. (J) *Cpt1a* and (K) *Dio1* mRNA expression in mouse primary hepatocytes. (L) Expression of fibrotic markers in primary hepatic stellate cells (PmHSCs) isolated from WD‐fed mice, with or without Resmetirom treatment. HPRT or GAPDH was used to normalize mRNA levels. Data are presented as mean ± SEM. Statistical significance was determined using a *t*‐test or a one‐way ANOVA followed by Bonferroni's multiple comparison test, with significance levels indicated as follows: *p < 0.05, **p < 0.01, ***p < 0.001.

Based on these results, we selected the 3 mg/kg dose for subsequent in vivo experiments. As anticipated, mice on WD displayed expected increases in weight gain and food intake relative to those on a chow diet; however, Resmetirom administration did not induce any significant changes in these parameters (Figure 1A,B). Circulating levels of liver function markers, including ALT and AST, were elevated in WD‐fed mice but returned to baseline levels following Resmetirom treatment (Figure 1C). A comparable reduction was detected for serum cholesterol concentrations (Figure 1D). Biochemical analyses of hepatic tissue demonstrated that Resmetirom significantly reduced liver weight and hepatic triglyceride content, both of which were elevated in WD‐fed mice (Figure 1E,F). As previously reported [25], protein levels of type 1 deiodinase (DIO1), the most abundant deiodinase enzyme in the liver, crucial for converting thyroxine (T4) into the active thyroid hormone triiodothyronine (T3), were increased in the liver of mice treated with Resmetirom (Figure 1G). Moreover, the histological assessment revealed a marked reduction in lipid deposition and collagen content in liver sections from Resmetirom‐treated mice (as shown by Oil Red O and Sirius Red staining, respectively) (Figure 1H). Furthermore, no significant differences were observed in Ki67 and cleaved caspase‐3 (CC3) staining, indicating no major alterations in proliferation or apoptosis. However, Resmetirom treatment was associated with a reduction in CD68‐positive areas, suggesting an improvement in hepatic inflammation (Figure S5A).

To further investigate the cellular mechanisms underlying the hepatoprotective effects of Resmetirom, primary hepatocytes and HSCs were isolated from these animals and analyzed separately. Oil Red O staining in primary hepatocytes confirmed the high lipid content in cells isolated from the livers of mice on WD, which was blunted by Resmetirom (Figure 1I). In agreement with previous publications [26], Resmetirom induced the expression of Dio1 and Cpt1a in hepatocytes (Figure 1J,K), supporting its role in enhancing fatty acid oxidation and contributing to the reduction in fat content. Furthermore, primary HSCs (PmHSCs) isolated from WD‐fed mice exhibited elevated expression of fibrotic markers, which was reduced by Resmetirom treatment (Figure 1L). Overall, these data indicate that in a mouse model of MASH, Resmetirom seems to exert beneficial effects in both hepatocytes and HSCs.

**FIGURE 2:**
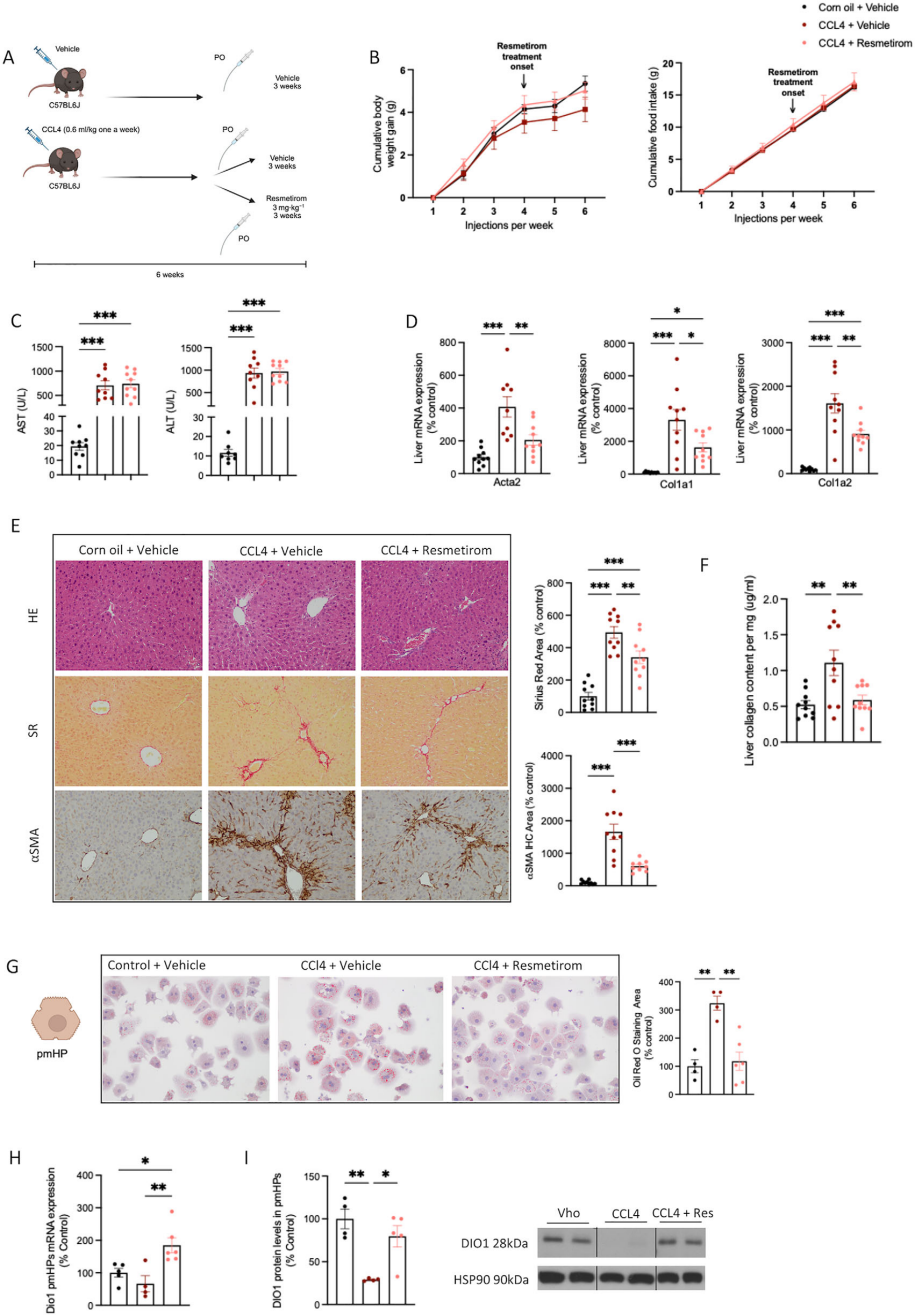
Resmetirom improves fibrosis in mice induced by CCL4. (A) Descriptive diagram of the mouse model experiment, in which mice were injected weekly with corn oil or 0.6 mL/kg of Carbon tetrachloride (CCL4) for 6 weeks, and oral administration of vehicle or Resmetirom for 3 weeks (*n* = 9–10 per group). (B) Cumulative body weight change and food intake. (C) Serum levels of AST and ALT in this mouse model. (D) mRNA expression of fibrosis markers in the liver of these mice. (E) Representative micrographs of Sirius Red staining (collagen deposition) and alpha‐SMA immunohistochemistry in liver sections. (F) Hepatic collagen content levels. (G) Representative micrographs of Oil Red O staining in primary hepatocytes (pmHP) isolated from CCL4‐treated mice, with or without Resmetirom treatment. (H) *Dio1* mRNA and (I) DIO1 protein levels in mouse primary hepatocytes. HPRT or GAPDH was used to normalize mRNA levels. Data are presented as mean ± SEM. Statistical significance was determined using a one‐way ANOVA followed by Bonferroni's multiple comparison test, with significance levels indicated as follows: *p < 0.05, **p < 0.01, ***p < 0.001.

We next evaluated the effect of Resmetirom in a nonobese model of liver fibrosis to determine whether it could exert beneficial effects independent of its action on fatty acid oxidation in hepatocytes. Mice were treated once weekly with 0.6 mL/kg CCl_4_ for 6 weeks, with Resmetirom administered orally during the last 3 weeks (Figure 2A). Body weight and food intake remained unchanged throughout the experiment (Figure 2B). As expected, serum transaminase levels were markedly elevated upon CCl_4_ treatment; however, Resmetirom did not reverse these elevations (Figure 2C). In contrast, the strong induction of fibrogenic genes in the liver of CCl_4_‐treated mice was significantly reduced by Resmetirom (Figure 2D). Consistently, Sirius Red staining, alpha‐smooth muscle actin (a‐SMA) staining, and liver collagen content, which were markedly increased in CCl_4_‐treated mice, were reduced in animals receiving Resmetirom (Figure 2E,F). Next, primary hepatocytes were isolated from these animals. Oil Red O staining in primary hepatocytes confirmed the low lipid content in cells isolated from the livers of mice treated with CCL_4_, which was blunted by Resmetirom (Figure 2G). Resmetirom also induced the mRNA expression and protein levels of DIO1 in primary hepatocytes of mice treated with CCL_4_ (Figure 2H,I). Taken together, these findings show that Resmetirom improves liver fibrosis regardless of the obese status of the mice, pointing to a potential direct effect on HSCs.

### 2.4 Resmetirom Attenuates TGF‐β1‐induced HSCs Activation via *THRα*

**FIGURE 3:**
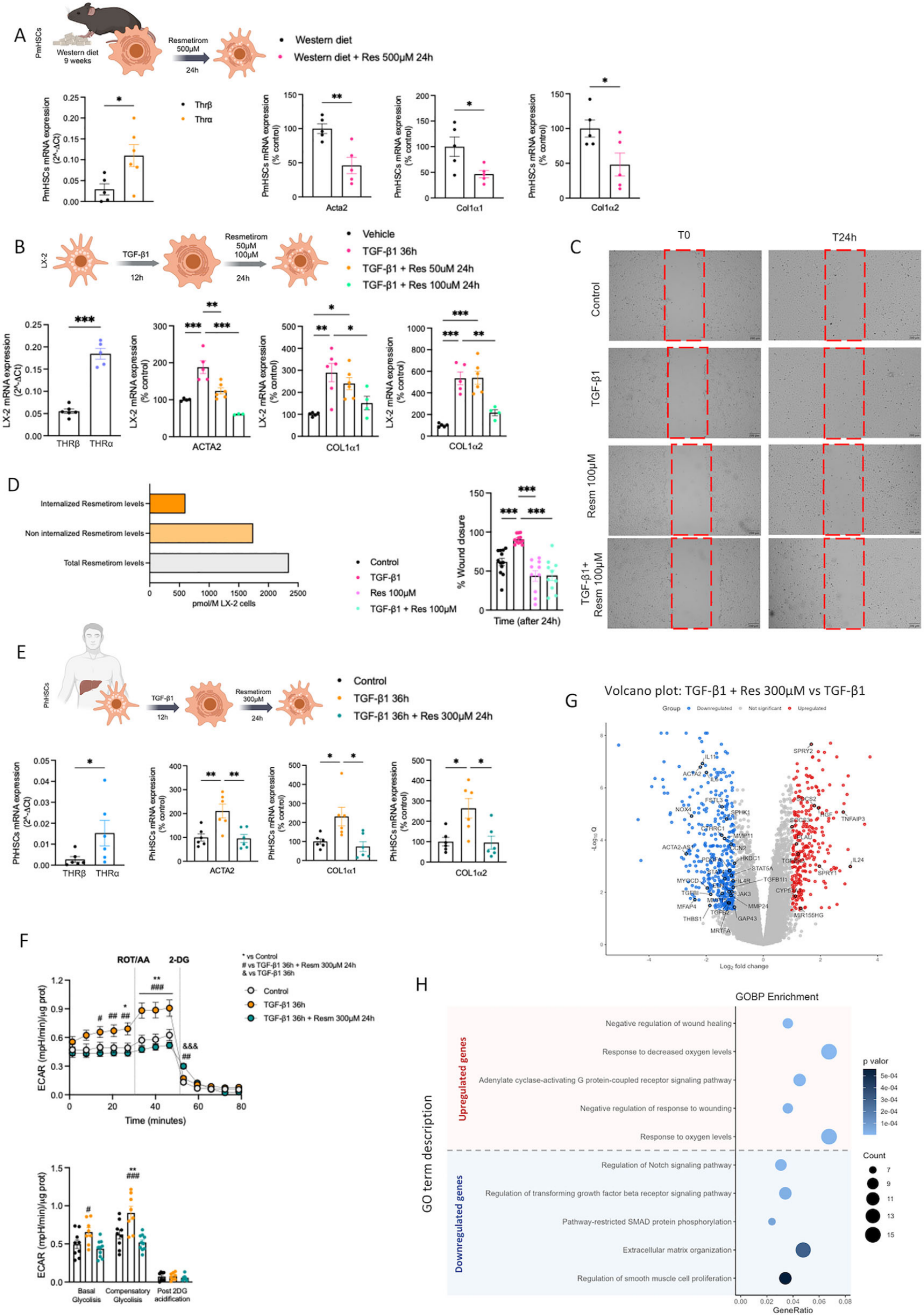
Resmetirom attenuates the activation of HSCs induced by TGF‐β1 or a Western diet. (A) *Thr𝛼* and *Thrβ* relative mRNA expression and fibrosis markers mRNA levels of PmHSCs isolated from mice fed a Western diet (WD) for 9 weeks, followed by an in vitro treatment with either a vehicle or Resmetirom (500 µM) for 24 h (*n* = 5–6 per group). (B) THR𝛼 and THRβ relative mRNA expression and fibrosis markers mRNA levels in LX‐2 cells treated with TGF‐β1 during 12 h alone, followed by a co‐treatment with either a vehicle or Resmetirom (50–100 µM) for 24 h (*n* = 5–6 per group). (C) Representative images and quantification of the scrape wound in LX‐2 generated in the cell layer and then treated with TGF‐β1 for 12 h alone, followed by a co‐treatment with either a vehicle or Resmetirom (100 µM) for 24 h (*n* = 10–12). (D) Intracellular accumulation of Resmetirom in LX‐2 cells treated with 100 µM resmetirom for 2 h, and the contribution of nonspecific binding. (E) THR𝛼 and THRβ relative mRNA expression and fibrosis markers mRNA levels in PhHSCs treated with TGF‐β1 during 12 h, followed by co‐treatment with either a vehicle or Resmetirom (300 µM) for 24 h (*n* = 5–6 per group). (F) ECAR in phHSCs, subjected to the same treatment conditions as described in (E) (*n* = 9–10 per group). (G) Volcano plot showing differentially expressed genes in PhHSCs treated with TGF‐β1 36 h alone versus those co‐treated with TGF‐β1 36 h and Resmetirom (300 µM) for 24 h. Upregulated genes (red) and downregulated genes (blue) were identified with a cutoff of |log_2_FC| > 1 and adjusted *p*‐value < 0.05 (*n* = 4 per group). (H) Gene Ontology Biological Process (GOBP) enrichment analysis of differentially expressed genes from (G), showing upregulated pathways (red) and downregulated pathways (blue) in PhHSCs co‐treated with Resmetirom and TGF‐β1 compared with TGF‐β1 alone. HPRT or GAPDH was used to normalize mRNA levels. Data are presented as mean ± SEM. Statistical significance was determined using a *t*‐test or a one‐way ANOVA followed by Bonferroni's multiple comparison test, with significance levels indicated as follows: *p < 0.05, **p < 0.01, ***p < 0.001.

Given that both *THRβ* and *THRα* are expressed in HSCs and that Resmetirom demonstrated beneficial effects in both hepatocytes and HSCs from mice fed a WD, our initial step was to measure the expression of both receptors in PmHSCs, the human LX‐2 line, and primary human HSCs (PhHSCs). The results revealed that in all these cell types, there was a higher expression of *THRα* compared with *THRβ* (Figure 3A,B,E).

Following this, our next objective was to investigate whether Resmetirom could exert a direct effect on HSCs. To this end, we isolated primary HSCs from mice fed a Western diet for 9 weeks, and once plated, these HSCs were treated with Resmetirom for 24 h. The results showed that Resmetirom reduced the gene expression of fibrotic markers such as *Acta2, Colα1*, and *Col1α2* (Figure 3A). Then, we assessed the mRNA expression of fibrotic markers in LX‐2 cells treated with TGF‐β1 in the presence or absence of Resmetirom for 24 h. Similar to the findings in PmHSCs, the compound attenuated the expression of TGF‐β1‐induced fibrotic markers in a dose‐dependent fashion (Figure 3B).

To extend our analysis of Resmetirom's effects on our HSCs cell line, we investigated its role in regulating the wound‐healing and migratory capacities of LX‐2 cells. These properties were studied in collagen‐precoated culture plates. A wound was created in the monolayers of LX‐2 cells. While TGF‐β1 treatment induced a significant increase in cell migration, Resmetirom effectively reduced this effect (Figure 3C). Importantly, this effect was independent of changes in cell viability or proliferation, as demonstrated by crystal violet staining or cell proliferation assays, respectively (Figure S6A,B). Furthermore, apoptosis was not affected by Resmetirom treatment, since protein levels of cleaved caspases 3 and 7 were unchanged (Figure S6C).

To determine whether HSCs can incorporate Resmetirom, intracellular levels of the compound were quantified in LX‐2 cells treated with 100 µM Resmetirom for 2 h. Appropriate control conditions were included to correct for nonspecific binding and extraction efficiency. All samples were analyzed by UPLC–MS/MS. After correction, a net intracellular accumulation of 601.8 pmol/10^6^ cells was detected. Notably, the signal detected in the cell‐free control revealed substantial nonspecific binding of resmetirom to the cell culture plastic (525.5 pmol) (Figure 3D). These findings indicate that Resmetirom is effectively taken up by LX‐2 cells, while also exhibiting a marked tendency to bind nonspecifically to plastic surfaces, likely due to its high lipophilicity.

To validate the potential clinical significance of our results, we performed the same experiment using PhHSCs, where, once again, Resmetirom mitigated the fibrotic effect induced by TGF‐β1 (Figure 3E). Finally, since it is well established that activated HSCs exhibit a highly glycolytic phenotype [16], we investigated the potential effect of Resmetirom on this metabolic process. Indeed, TGF‐β1 increased the extracellular acidification rate (ECAR), which is commonly used as an indicator of glycolysis (Figure 3F). When these cells were co‐treated with Resmetirom, the drug exhibited an inhibitory effect on ECAR (Figure 3F). However, no significant differences were observed among the three experimental groups in the oxygen consumption rate (OCR), a key parameter for assessing mitochondrial respiration (Figure S6D).

To further corroborate these results, we performed RNA sequencing analysis comparing PhHSCs treated with TGF‐β1 alone to those co‐treated with Resmetirom and TGF‐β1. This analysis revealed 273 upregulated and 426 downregulated genes, as shown in the volcano plot (Figure 3G). Among the upregulated genes, we identified several involved in the negative regulation of wound healing and the response to oxygen levels. Conversely, the downregulated genes included key components of the Notch signaling pathway, TGF‐β1 signaling, and extracellular matrix organization, among others, as illustrated by the Gene Ontology Biological Process (GOBP) enrichment (Figure 3H).

**FIGURE 4:**
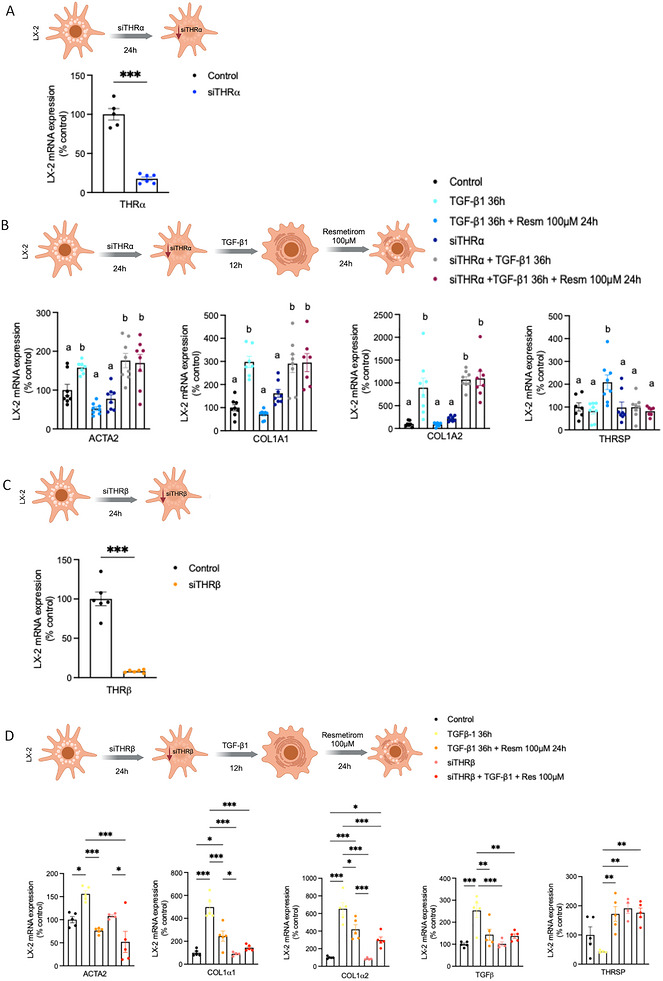
Resmetirom attenuates the activation of HSCs through its selective action on the thyroid hormone receptor alpha. (A) THR𝛼 mRNA levels in LX‐2 cells transfected with si0 or siTHR𝛼. (B) mRNA levels of fibrosis markers in LX‐2 cells transfected with si0 or siTHR𝛼, treated with TGF‐β1 for 12 h alone, followed by a co‐treatment with either a vehicle or Resmetirom (100 µM) for 24 h (*n* = 5–12 per group). (C) THRβ mRNA levels in LX‐2 cells transfected with si0 or siTHRβ. (D) mRNA levels of fibrosis markers in LX‐2 cells transfected with si0 or siTHRβ, treated with TGF‐β1 for 12 h alone, followed by a co‐treatment with either a vehicle or Resmetirom (100 µM) for 24 h (*n* = 5–12 per group). HPRT or GAPDH was used to normalize mRNA levels. Data are presented as mean ± SEM. Statistical significance was determined using a *t*‐test or, when data followed a normal distribution, a one‐way ANOVA followed by Bonferroni's multiple comparison test; when data did not meet normality assumptions, the Kruskal–Wallis test was applied, followed by Dunn's multiple comparisons test. Significance levels were indicated as follows: *p < 0.05, **p < 0.01, ***p < 0.001. In (B), different letters denote statistically significant differences between the groups.

Subsequently, we proceeded to assess the function of *THRα* and *THRβ* in regulating the effects of Resmetirom on the activation of HSCs. To this end, we selectively silenced *THRα* or *THRβ* in LX‐2 cells (Figure 4A,C) and then treated the cells with TGF‐β1, with or without Resmetirom. The incubation with TGF‐β1 resulted in the predicted rise of the gene expression of fibrotic markers, which was attenuated upon Resmetirom treatment. However, genetic silencing of THRs differentially affected the action of Resmetirom. Specifically, silencing *THRα* prevented Resmetirom from ameliorating TGF‐β1‐induced activation of HSCs (Figure 4B). In contrast, silencing *THRβ* did not affect the capacity of Resmetirom to mitigate the effects of TGF‐β1 (Figure 4D). These findings suggest that Resmetirom requires intact *THRα* signaling to exert its beneficial action in HSCs.

### 2.5 Hepatocyte and HSC SLCO1β1 Overexpression Lowers the Effective Resmetirom Dose

**FIGURE 5:**
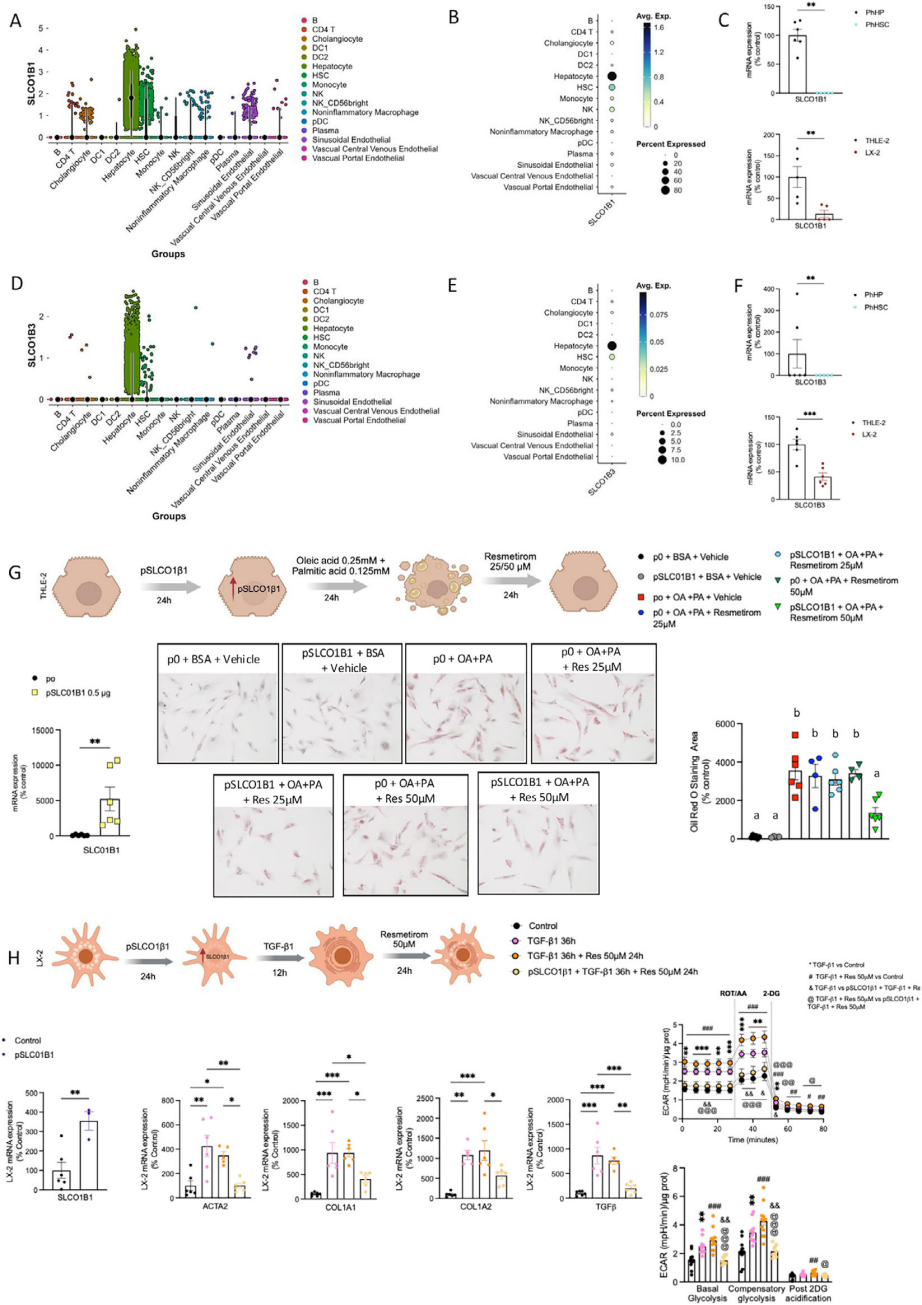
Overexpression of SLCO1β1 in hepatocytes and HSCs diminishes Resmetirom dose. GeyserPlot (A) and DotPlot (B) showing the expression of SLCO1β1 across the different hepatic cell types of the analyzed scRNA‐seq dataset. The scRNA‐seq dataset is composed of healthy human (*n* = 4) and cirrhotic livers (*n* = 2) from the public datasets (GSE185477and GSE174748). (C) mRNA expression of SLCO1β1 in primary human hepatocytes and HSCs and in THLE‐2 and LX‐2 (*n* = 6). GeyserPlot (D) and DotPlot (E) showing the expression of SLCO1β3 across the different cell types of the analyzed scRNA‐seq dataset. (F) mRNA expression of SLCO1β3 in primary human hepatocytes and HSCs and in THLE‐2 and LX‐2 (*n* = 6). (G) Oil Red O staining in THLE‐2 cells, with or without SLCO1β1 overexpressed, following treatment with oleic acid (OA, 0.25 mM) and palmitate (PA, 0.125 mM) for 12 h, and subsequent co‐exposure to either a vehicle or Resmetirom (25 or 50 µM) for 24 h (*n* = 6 per group). (H) Expression of fibrotic markers and ECAR in LX‐2 cells with and without the overexpression of SLCO1β1, followed by a treatment with TGF‐β1 for 12 h and a subsequent co‐exposure to either a vehicle or Resmetirom (50 µM) for 24 h (*n* = 6 per group). HPRT was used to normalize mRNA levels. Data are presented as mean ± SEM. Statistical significance was determined using one‐way ANOVA followed by Bonferroni's multiple comparison test, with significance levels indicated as follows: *p < 0.05, **p < 0.01, ***p < 0.001. In Figure 5G, different letters denote statistically significant differences between the groups.

Since the transport of Resmetirom has been shown to depend on the solute carrier organic anion transporter family member 1B1 (SLCO1β1, also named OATP1B1) [25], we next examined the expression of this gene in human hepatic cell populations to better understand its cellular uptake and liver specificity. To this end, we analyzed the single‐cell RNA‐seq datasets described above (GEO accessions: GSE185477 and GSE174748) [19, 20]. SLCO1B1 is highly expressed in hepatocytes, but its expression is lower in HSCs and natural killer cells (Figure 5A,B). These findings were further validated by real‐time PCR in primary human hepatocytes and HSCS, as well as in THLE‐2 and LX‐2 cells (Figure 5C).

We also examined the expression of SLCO1β3, another hepatic organic anion transporter, across the different hepatic cell types of the analyzed scRNA‐seq dataset (Figure 5D,E) and by real‐time PCR in primary human hepatocytes and HSCS and in THLE‐2 and LX‐2 cells (Figure 5F). The results revealed that SLCO1β3 follows a similar distribution pattern across cell types; however, its expression levels were consistently lower than those observed for SLCO1β1, highlighting SLCO1B1 as the predominant transporter in hepatocytes.

Overall, SLCO1β1 expression was always much higher in hepatocytes and THLE‐2 cells, whereas it remained low in HSCs. This marked differential expression of SLCO1β1 in hepatocytes and HSCs may explain the cell‐type‐specific differences in Resmetirom sensitivity, with effective doses of 100 µM for primary human hepatocytes versus 500 and 300 µM for primary mouse and human HSCs, respectively.

We next investigated whether genetic overexpression of SLCO1β1 could reduce the effective dose of Resmetirom. Hepatocytes were cotreated with a palmitate/oleate mixture and Resmetirom at two different low doses (25 and 50 µM), in the presence or absence of SLCO1β1. Our results indicate that the previously subeffective dose of Resmetirom (50 µM; Figure S2A) became effective upon ectopic expression of SLCO1β1, as shown by the reduction in Oil red O staining (Figure 5G).

We performed a similar experiment in LX‐2 cells, cotreating them with TGF‐β1 and a low dose of Resmetirom (50 µM) in the presence of SLCO1β1 overexpression. We found that this previously ineffective dose (see dose–response in Figure 3B) became effective, as indicated by reduced expression of several fibrotic markers and decreased glycolytic rate (Figure 5H).

To compare with Resmetirom, LX‐2 cells were treated with the THRα agonist CO23 [27]. As expected, cells treated with CO23 (0.5 nM) exhibited markedly increased expression of THRSP and DIO1 (Figure S7A). When co‐administered with TGF‐β1, CO23 reduced fibrotic marker expression to a similar extent as Resmetirom (100 µM) (Figure S7B), without affecting cell proliferation or viability (Figure S7C,D). These results indicate that very low doses of THRα agonists are highly effective in attenuating HSCs activation, in contrast to the substantially higher doses required for Resmetirom.

### 2.6 Resmetirom Increases Fatty‐Acid Amide Hydrolase (FAAH) in HSCs

**FIGURE 6:**
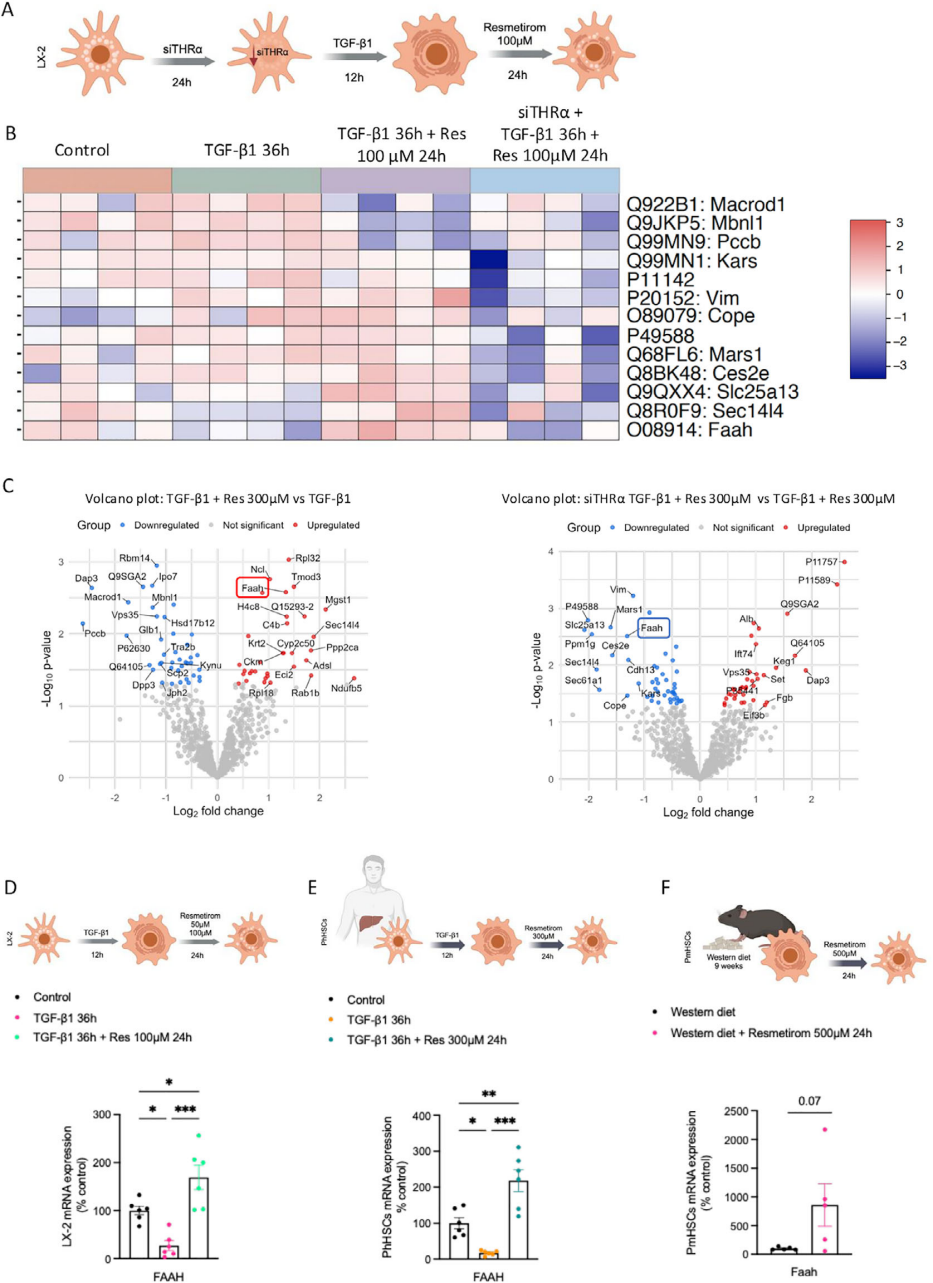
Resmetirom attenuates the activation of HSCs through its selective action on the thyroid hormone receptor alpha and FAAH. (A) Experimental design for proteomic analysis performed in LX‐2 cells transfected with si0 or siTHR𝛼, treated with and without TGF‐β1 for 12 h alone, followed by a co‐treatment with either a vehicle or Resmetirom (100 µM) for 24 h. (B) Heatmap of the upregulated and downregulated proteins in the four experimental conditions. Volcano plots of protein expression determined by LC‐MS/MS proteomics of (C) TGF‐β1 and Resmetirom‐treated LX‐2 cells compared with TGF‐β1‐treated LX‐2 cells (*n* = 4 per group), or LX‐2 cells knockdown for THR𝛼 and treated with TGF‐β1 and Resmetirom compared with TGF‐β1 and Resmetirom‐treated LX‐2 cells (*n* = 4 per group). Values obtained for FAAH are represented in the graph. (D) mRNA levels of FAAH in LX‐2 cells treated with TGF‐β1 during 12 h alone, followed by a co‐treatment with either a vehicle or Resmetirom (100 µM) for 24 h, with (*n* = 5–6 per group). (E) mRNA levels of FAAH in primary human HSCs treated with TGF‐β1 during 12 h alone, followed by a co‐treatment with either a vehicle or Resmetirom (100 µM) for 24 h (*n* = 5–6 per group). (F) FAAH mRNA levels in primary murine HSCs isolated from mice fed a Western Diet (WD) for 9 weeks, followed by an in vitro treatment with either a vehicle or Resmetirom (500 µM) for 24 h (*n* = 5–6 per group). HPRT or GAPDH was used to normalize mRNA levels. Data are presented as mean ± SEM. Statistical significance was determined using a *t*‐test or a one‐way ANOVA followed by Bonferroni's multiple comparison test, with significance levels indicated as follows: *p < 0.05, **p < 0.01, ***p < 0.001.

To investigate the protein changes underlying the effects of Resmetirom in HSCs, we conducted a proteomic analysis in LX‐2 cells with silenced *THRα* followed by treatment with TGF‐β1 in the presence or absence of Resmetirom (Figure 6A). Among the proteins modulated by TGF‐β1 (Figure 6B), one of the most significantly altered proteins was FAAH, a membrane‐associated enzyme that hydrolyzes the endocannabinoid anandamide (AEA) and, to a lesser extent, 2‐arachidonoylglycerol [28, 29]. Notably, multiple studies have implicated the endocannabinoid system in metabolic diseases, including MASLD [30].

FAAH protein levels were significantly reduced by TGF‐β1treatment; however, Resmetirom administration restored its expression. Interestingly, FAAH levels remained unchanged when *THRα* was silenced. Volcano plot analysis further demonstrated that while FAAH expression was increased in cells treated with both TGF‐β1 and Resmetirom compared with those treated with TGF‐β1 alone, this effect was entirely abolished in the absence of *THRα* (Figure 6C). These findings suggest that Resmetirom induces FAAH expression in a THRα‐dependent manner.

The proteomic results were further corroborated by measuring gene expression of *FAAH* in LX‐2 cells and PhHSCs activated with TGF‐β1. As expected, TGF‐β1 treatment significantly reduced *FAAH* expression, whereas Resmetirom triggered its expression (Figure 6D,E). Also, PmHSCs isolated from mice fed a western diet for 9 weeks and treated with Resmetirom displayed higher levels of *Faah* (Figure 6F).

### 2.7 The THRα‐FAAH Pathway Mediates the Effects of Resmetirom in HSCs

**FIGURE 7:**
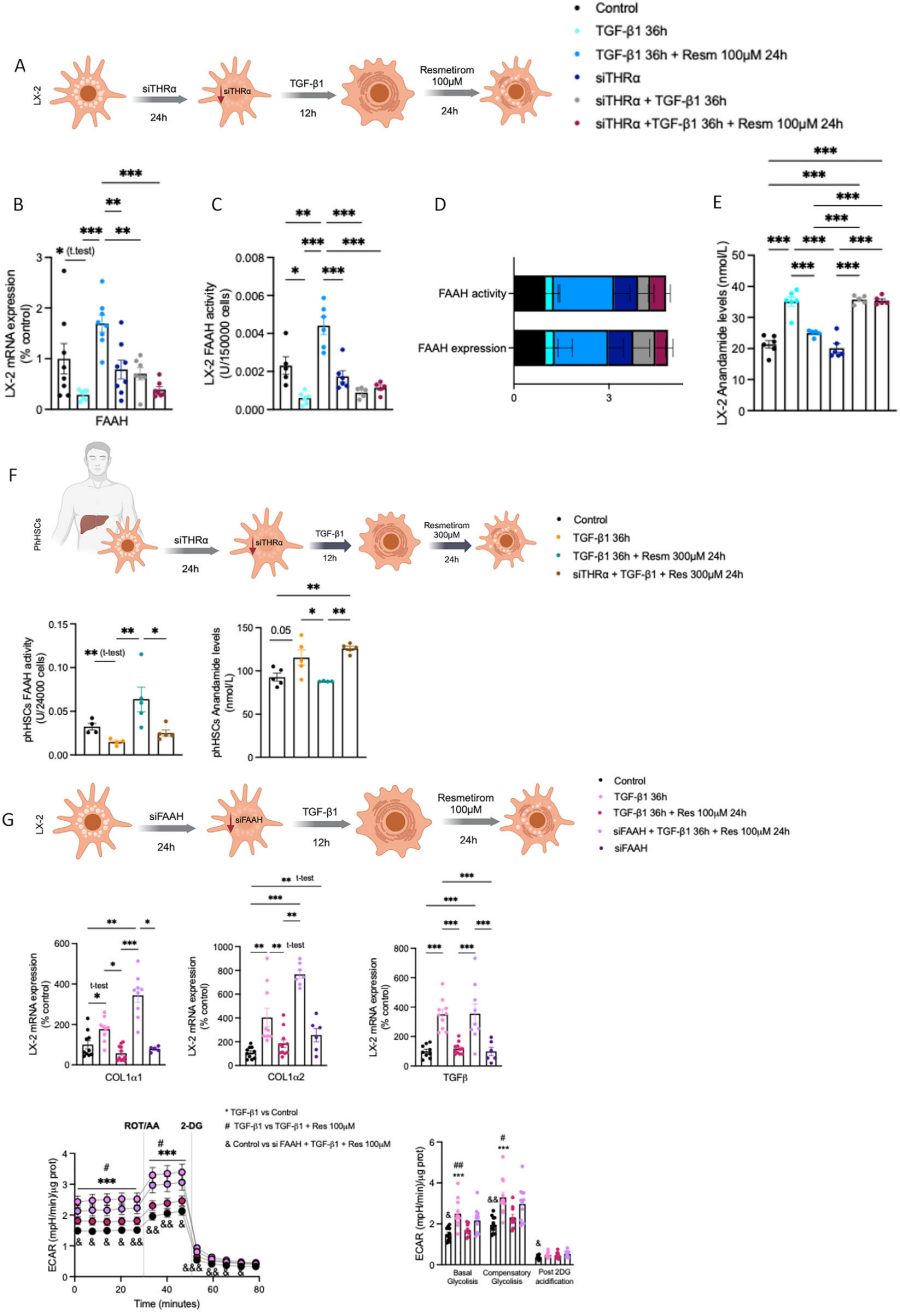
Resmetirom requires FAAH to reduce HSC activation. (A) Experimental design done in LX‐2 cells transfected with si0 or siTHR𝛼, treated with and without TGF‐β1 during 36 h, followed by treatment with either a vehicle or Resmetirom (100 µM) for 24 h. (B) mRNA levels, (C) activity of FAAH, (D) FAAH activity and expression relation, and (E) AEA content in LX‐2 cells transfected with si0 or siTHR𝛼, treated with and without TGF‐β1 during 12 h alone, followed by a co‐treatment with either a vehicle or Resmetirom (100 µM) for 24 h. (*n* = 4–12 per group). (F) Activity of FAAH and AEA content in phHSCs transfected with si0 or siTHR𝛼, treated with and without TGF‐β1 during 12 h alone, followed by a co‐treatment with either a vehicle or Resmetirom (300 µM) for 24 h (*n* = 4 per group). (G) mRNA expression of fibrosis markers and ECAR in LX‐2 cells transfected with si0 or siFAAH, treated with and without TGF‐β1 during 36 h, followed by treatment with either a vehicle or Resmetirom (100 µM) for 24 h. (*n* = 9–16 per group). HPRT or GAPDH was used to normalize mRNA levels. Data are presented as mean ± SEM. Statistical significance was determined using a *t*‐test or a one‐way ANOVA followed by Bonferroni's multiple comparison test, with significance levels indicated as follows: *p < 0.05, **p < 0.01, ***p < 0.001.

Given the strong reduction in both *FAAH* gene expression and FAAH protein levels following TGF‐β1 treatment, and their subsequent restoration by Resmetirom, we assessed whether this effect was mediated by THRα (Figure 7A). Resmetirom was able to induce *FAAH* expression in LX‐2 cells pretreated with TGF‐β1; however, this effect was abolished when THRα was silenced (Figure 7B). In line with the transcriptional data and protein data, FAAH enzymatic activity also followed the same trend (Figure 7C). For instance, there was a clear correlation between *FAAH* gene expression and activity (Figure 7D). Furthermore, levels of AEA inversely mirrored FAAH activity; lower FAAH expression led to AEA accumulation, while Resmetirom reduced AEA content through FAAH activation (Figure 7E). Similar results were obtained in PhHSCs, where both FAAH activity and AEA content followed the same pattern as in LX‐2 cells (Figure 7F). Next, we aimed to determine the causal role of FAAH in mediating the actions of Resmetirom. To this end, *FAAH* was silenced in LX‐2 cells before treatment with TGF‐β1, in the presence or absence of Resmetirom. While Resmetirom effectively attenuated the TGF‐β1‐induced upregulation of fibrogenic markers and glycolytic activity, this protective effect was lost upon *FAAH* knockdown (Figure 7G). These findings suggest that FAAH is essential for the THRα‐mediated antifibrotic actions of Resmetirom in HSCs.

## 3 Discussion

Liver fibrosis is a progressive disease with limited treatment options, often leading to severe complications such as cirrhosis and liver failure. In this study, we report a novel molecular mechanism underlying the therapeutic effects of Resmetirom, the current pipeline therapeutic strategy, in ameliorating liver fibrosis. While the primary action of Resmetirom involves targeting hepatocytes, our findings reveal an additional role in directly modulating HSC activity. Specifically, Resmetirom suppresses HSC activation by downregulating profibrogenic pathways, thereby reducing extracellular matrix deposition and collagen synthesis.

Notably, the mechanism of Resmetirom differs between hepatocytes and HSCs. While in hepatocytes, Resmetirom acts primarily through THRβ, the predominant isoform in these cells, in HSCs, it exerts its actions via THRα, the primary isoform expressed in HSCs [15]. The activation of THRβ triggers the expression of lipid‐metabolizing genes [31], enhances mitochondrial function and fatty acid oxidation [26, 32], and promotes mitochondrial biogenesis and function by increasing the expression of deiodinase 1 (DIO1) and malic enzyme 1 (ME1) [26]. Consistent with these findings, our hepatocyte experiments showed that genetic deletion of THRβ abolished all beneficial effects of Resmetirom in this cell type. Interestingly, in a liver fibrosis model without metabolic syndrome (CCl_4_‐treated mice), Resmetirom was still able to ameliorate fibrosis. This observation suggests that Resmetirom may attenuate liver fibrosis through THRα‐dependent mechanisms independent of its well‐characterized actions on mitochondrial function.

Although Resmetirom exhibits 28‐fold greater selectivity for THRβ over THRα due to its molecular structure, it does retain some activity for the alpha receptor [14, 25]. In cell‐based luciferase reporter gene assays, Resmetirom (MGL‐3196) demonstrated activation of both THRβ and THRα, with EC50 values of 3.11 and 149.0 µM, respectively [33]. This indicates that while Resmetirom is highly selective for THRβ, it still has some activity on THRα, albeit at much higher concentrations. Notably, concentrations of Resmetirom in the liver are approximately four times higher than those in serum, thereby enhancing its ability to bind to THRs [33]. Importantly, both *Thrβ* and *Thrα* expression is strongly repressed during liver injury, and the treatment of primary mouse HSCs with T3 attenuates TGF‐β1 signaling in HSC in a THRα‐dependent manner [15]. Therefore, the activation of THRα by T3 ameliorates fibrogenesis in HSCs. Our results demonstrate that Resmetirom can activate THRα in HSCs and induce effects similar to those reported for T3, including the suppression of TGF‐β1 signaling and the reduction in the expression of fibrotic markers. These effects were exclusively mediated by THRα, as genetic silencing of THRα completely abolished the antifibrotic actions of Resmetirom. In contrast, the knockdown of THRβ in HSCs did not affect the ability of Resmetirom to alleviate TGF‐β1 signaling or reduce the expression of fibrotic markers. This highlights the critical role of THRα in mediating the actions of Resmetirom in HSCs, independent of its established selectivity for THRβ in other cell types, such as hepatocytes. Notably, preclinical and clinical studies have demonstrated that Resmetirom does not influence bone mineral density, cardiac alpha myosin heavy chain (MHC) expression in thyroidectomized rats, or heart rate—effects typically mediated by THRα [14, 34, 35]. However, as a liver‐targeted drug, Resmetirom facilitates selective binding and activation of THRα within hepatic cells.

In addition to its higher specificity for the THRβ isoform, another key determinant of Resmetirom activity is the transporter SLCO1β1 [25]. Analysis of human liver scRNA‐seq data revealed that this transporter is highly expressed in hepatocytes but is almost undetectable in HSCs. The absence of SLCO1β1 in HSCs may explain why the effective doses of Resmetirom required in this cell type are higher than those in hepatocytes. Indeed, our experiments demonstrated that overexpression of SLCO1β1 in both hepatocytes and HSCs markedly reduced the effective doses of Resmetirom, suggesting that agonists of this transporter might enhance the efficacy of Resmetirom. Notably, comparison with the selective THRα agonist CO23 further supports this interpretation, as CO23 was able to elicit antifibrotic effects at doses several orders of magnitude lower than those required for Resmetirom.

Our proteomic analysis revealed several novel protein targets modulated by Resmetirom, among which FAAH emerged as a key focus. FAAH is the primary enzyme responsible for hydrolyzing anandamide, a bioactive endocannabinoid. This finding holds particular significance given the well‐documented role of endocannabinoid signaling in driving the activation of quiescent HSCs into myofibroblast‐like cells [36, 37, 38]. While prior research has predominantly focused on cannabinoid receptor 1 (CB1)—with evidence supporting the therapeutic potential of CB1 antagonism in MASLD [39]—recent studies have challenged this paradigm [40]. Notably, our data align with established observations that activated HSCs lack *FAAH* expression, resulting in reduced *Faah* levels in murine models of bile duct ligation (BDL) and carbon tetrachloride (CCl_4_)‐induced fibrosis. This FAAH deficiency promotes endocannabinoid accumulation, which exacerbates oxidative stress (ROS) and hepatocellular damage [41]. The diminished expression and activity of FAAH detected in activated HSCs were significantly restored by Resmetirom in a THRα‐dependent manner. Notably, changes in FAAH activity exhibited an inverse correlation with the levels of AEA, the endocannabinoid hydrolyzed by this enzyme. Specifically, while TGF‐β1 elevated AEA levels in HSCs under conditions of reduced FAAH expression and activity, Resmetirom effectively decreased AEA levels by enhancing FAAH activity.

Moreover, genetic silencing of FAAH attenuated the beneficial effects of Resmetirom on HSCs, suggesting its critical role as a mediator of the action of Resmetirom. Specifically, the inhibition of FAAH impaired the capacity of Resmetirom to reduce both the expression of fibrotic markers and the elevated glycolytic activity characteristic of activated HSCs. The identification of FAAH modulation by Resmetirom introduces a new dimension to its mechanism of action, potentially linking thyroid hormone receptor activity with endocannabinoid metabolism in HSC biology. This dual‐cell targeting mechanism, combining hepatocyte‐directed metabolic reprogramming and direct antifibrotic effects on HSCs, addresses both the metabolic and fibrotic components of MASH. This might provide a more comprehensive explanation for the efficacy of Resmetirom in clinical trials, where it demonstrated significant fibrosis improvement alongside MASH resolution [12, 13].

Although our data consistently support the activation of THRα as the primary mechanism mediating the response in HSCs, the effective doses used exceed the reported EC50 values for receptor engagement. This likely reflects the low expression of the hepatic transporter SLCO1β1 in HSCs, which may limit intracellular drug availability and necessitate higher extracellular concentrations. In this context, the use of elevated doses raises the possibility that off‐target effects could contribute, at least in part, to the observed responses. Nonetheless, the convergent genetic and mechanistic evidence presented here strongly supports a central role of THRα‐dependent signaling in mediating the antifibrotic actions of Resmetirom in HSCs, while not completely excluding the involvement of complementary pathways.

In summary, our study reveals that: (i) the antifibrotic action of Resmetirom is partially mediated through HSCs, (ii) while Resmetirom signals via THR‐β in hepatocytes, its effects in HSCs are mediated through THRα; and (iii) the presence of the enzyme FAAH in HSCs is critical for the efficacy of Resmetirom. These findings may support the development of future therapeutic strategies targeting THRα in HSC, in combination with existing approaches targeting hepatocyte THRβ.

## 4 Materials and Methods

### 4.1 Animals

Animal experiments were conducted in accordance with the standards approved by the Faculty Animal Committee at the University of Santiago de Compostela, and the experiments were performed in agreement with the Rules of Laboratory Animal Care and International Law on Animal Experimentation.

### 4.2 Animals and Diets

Mouse experiments were conducted according to the standard protocols approved by the Animal Ethics Committee at the University of Santiago de Compostela, and mice received the highest levels of humane care. C57BL/6J mice were housed in rooms under

 constant temperature (22 C), with a 12:12 h light/dark cycle. When the mice were 8 weeks old, they were given ad libitum access to a Western diet (TD88137; 42 % kJ fat, 15% kJ protein, 43% kJ carbohydrates, and 1.25% cholesterol, Ssniff) and its recommended chow diet (CD88137; 13% kJ fat, 18% kJ protein, 69% kJ carbohydrates, Ssniff) for 8 weeks. The macronutrient composition of each diet is indicated in Table S1. Food intake and body weight were measured weekly during all experimental phases. After the animals were euthanized, serum and liver samples were collected. All molecular and histological studies were conducted using fragments from the left lateral lobe.

### 4.3 Metabolic Flux Assays

Mitochondrial respiration and glycolytic activity were assessed by measuring the oxygen consumption rate (OCR) and extracellular acidification rate (ECAR), respectively, using high‐resolution respirometry with the Seahorse Bioscience XFe96 Extracellular Flux Analyzer (Agilent Technologies). For these experiments, 1.5 × 10^4^ cells were plated per well in an XFe96 cell culture microplate (102418‐000, Seahorse Bioscience, Agilent Technologies).

Before the assay, the culture medium was removed and replaced with prewarmed assay medium. This medium consisted of Seahorse XF DMEM (103575‐100, Seahorse Bioscience, Agilent Technologies) supplemented with 1 mM sodium pyruvate, 2 mM L‐glutamine, and 10 mM glucose. Cells were then incubated at 37°C under ambient air conditions.

For OCR determination, five baseline measurements were performed, and specific mitochondrial respiration modulators were sequentially introduced into the wells: oligomycin (1.5 µM), carbonyl cyanide‐4‐(trifluoromethoxy) phenylhydrazone (FCCP, 1 µM), and a combination of rotenone and antimycin A (0.5 µM) from the Cell Mito Stress Test Kit (103015‐100, Seahorse Bioscience, Agilent Technologies). Based on the manufacturer's guidelines (103015‐100, Seahorse Bioscience, Agilent Technologies), the following mitochondrial function parameters were calculated: basal respiration, ATP‐linked respiration, proton leak, maximal respiration, and nonmitochondrial oxygen consumption. For the assessment of ECAR, five baseline measurements were recorded before the sequential addition of glycolytic modulators. The first injection consisted of rotenone/antimycin (5 mM), followed by a second injection of 2‐deoxy‐D‐glucose (2‐DG, 500 mM), both from the glycolytic rate kit (103344‐100, Seahorse Bioscience, Agilent Technologies). Following the manufacturer's recommendations, the following glycolytic parameters were assessed: basal glycolysis, compensatory glycolysis, and post‐2‐DG acidification.

All OCR and ECAR values were normalized to protein content using the Bradford protein assay (Bio‐Rad Protein Assay Dye Reagent Concentrate, #5000006, Bio‐Rad).

### 4.4 Fatty Acid Amide Hydrolase Assay Kit

Enzymatic activity was measured using the MyBioSource kit (MBS2577859) following the manufacturer's instructions. Briefly, cells were homogenized in buffer (LX‐2, 100 µL; phHSCs, 50 µL) and centrifuged to obtain the supernatant. A standard curve (0–2 µmol/L) was prepared using serial dilutions of a 2 µmol/L working standard solution. Samples (20 µL) were added to 96‐well black plates in duplicate: one well for total activity (with 30 µL buffer) and another for nonspecific activity (with 30 µL inhibitor working solution). After a 5 min incubation at 25°C (light‐protected), 130 µL of freshly prepared substrate solution was added to each well. Plates were incubated at 37°C for 25 min, and fluorescence was measured at 340/440 nm. Specific activity was calculated by subtracting nonspecific from total fluorescence, and analyte concentrations were determined using the standard curve.

### 4.5 Statistical Analysis

Data are expressed as mean ± standard error of the mean (SEM). Statistical significance was determined by a two‐tailed Student's *t*‐test or Mann–Whitney test when two groups were compared, or one‐way ANOVA with Bonferroni posttest. The significance level was set at *p* < 0.05. GraphPad Prism Software 10.4.1 (532) was used to compute statistical analyses and graphics. See the detailed methods in the online supplemental material.

## Author Contributions

E.N. and A.C. conceived the study, designed and conducted experiments, analyzed results, and contributed to the manuscript preparation. B.L‐P., N.d.S.L., C.R., C.P., J.I., A.S., M.L.M‐C., A.F‐I., J.G‐S., S.B., C.D., M.V.‐R., V.P., M.S., and M.L. contributed to the performance of experiments and to the manuscript preparation. R.N. conceived the study; designed, coordinated, and supervised experiments; wrote the manuscript. All authors discussed the results, commented on the manuscript before submission, and agreed with the final submitted manuscript.

## Funding

This work was supported by grants from: FEDER/Ministerio de Ciencia, Innovación y Universidades‐Agencia Estatal de Investigación (RN: PID2021‐126096NB‐I00; MV‐R: PID2023‐152685OB‐I00 and CNS2024‐154920); Xunta de Galicia (RN: ED431C 2024/10, MV‐R: ED431C2023/09); Proyectos Investigación en Salud DTS20/00138 (to M.L.M.‐C.); Proyectos Investigación en Salud (MLMC: DTS20/00138). This research also received funding from the European Community's H2020 Framework Programme (ERC Synergy Grant‐2019‐WATCH‐ 810331, to R.N., V.P., and M.S.). The Centro de Investigación Biomédica en Red (CIBER) de Fisiopatología de la Obesidad y Nutrición (CIBERobn) and the Centro de Investigación Biomédica en Red (CIBER) de Enfermedades Hepáticas y Digestivas (CIBERehd) are initiatives of the Instituto de Salud Carlos III (ISCIII) of Spain, which is supported by FEDER funds.

## Ethics Statement

This study involves mice and was approved by the Faculty Animal Committee at the University of Santiago de Compostela (Number: 15012/2023/014).

## Conflicts of Interest

Miguel López is an Editorial Board member of MedComm and was not involved in the journal's review of or decisions related to this manuscript. He serves as scientific director of Gazella Biotech (https://gazellabiotech.com/). Rubén Nogueiras serves in the Advisory Board of Albor Biotech (https://alborbiotech.com/) and has received honoraria for conference presentations from Madrigal Pharmaceuticals. The remaining authors declare no conflicts of interest.

## Supporting information




**Supporting File 1**: mco270901‐supp‐0001‐SupMat.pdf

## Data Availability

The data that support the findings of this study are available from the corresponding author upon reasonable request.
