# Supplementary material for: Resmetirom Ameliorates Fibrogenesis in Hepatic Stellate Cells via Thyroid Hormone Receptor Alpha‐Fatty‐Acid Amide Hydrolase 1 Signaling Pathway
Source: MedComm (2020). 2026 Aug 5;7(8):e70901. doi: 10.1002/mco2.70901 (PMC13439220; doi:10.1002/mco2.70901)
Supplement: Supplementary file 1 [file MCO2-7-e70901-s001.pdf]

## Supplementary Figure 1

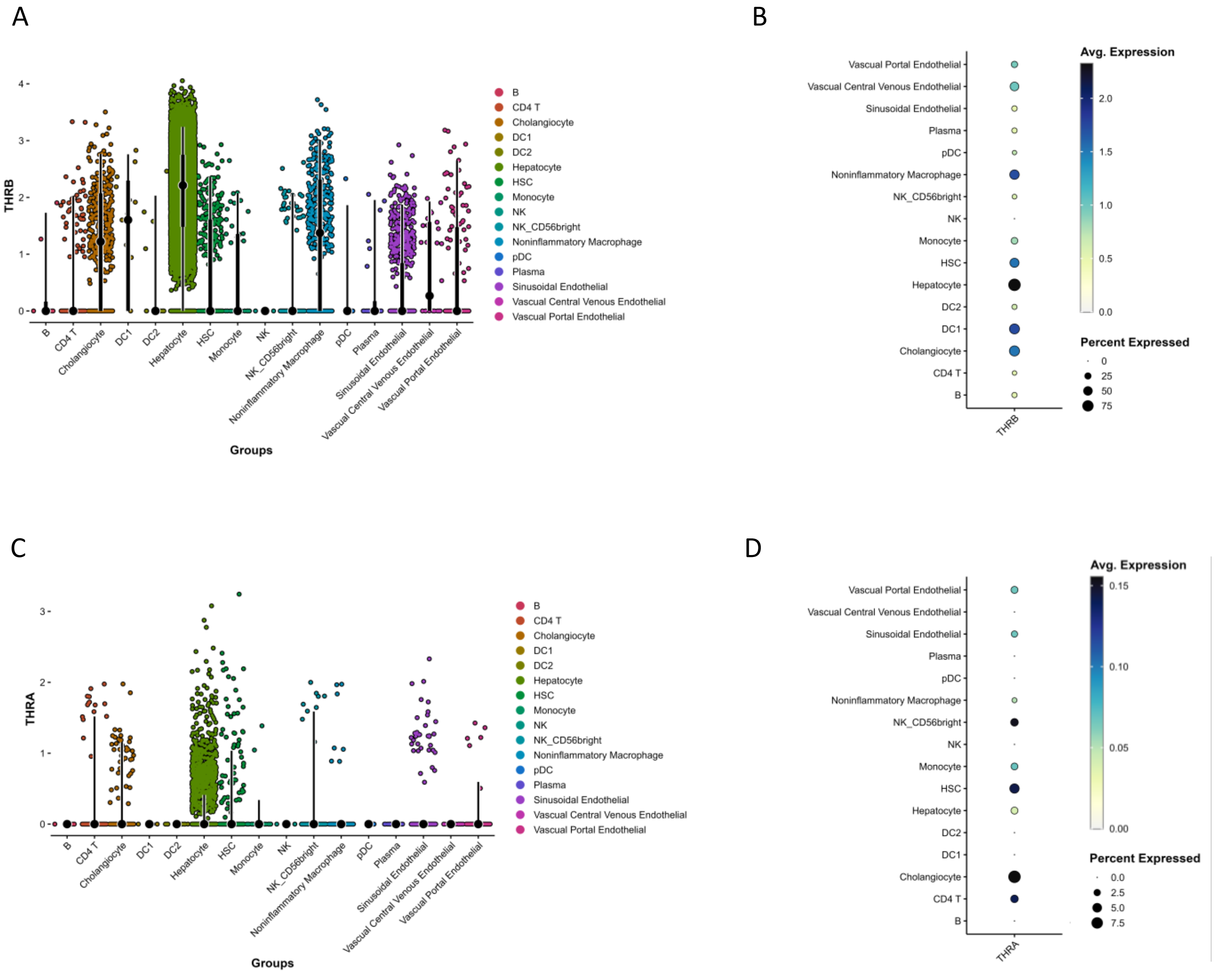


**Supplementary Figure 1. Expression of THRβ and THRα in the liver of people with MASLD/MASH** (A-B). GeyserPlot (A) and DotPlot (B) showing the expression of THRβ across the different cell types of the analyzed scRNA-seq dataset. GeyserPlot (C) and DotPlot (D) showing the expression of THRα different cell types of the scRNA-seq dataset. The scRNA-seq dataset is composed by healthy human (n=4) and cirrhotic livers (n=2) from the public datasets (GSE185477and GSE174748).

## Supplementary Figure 2

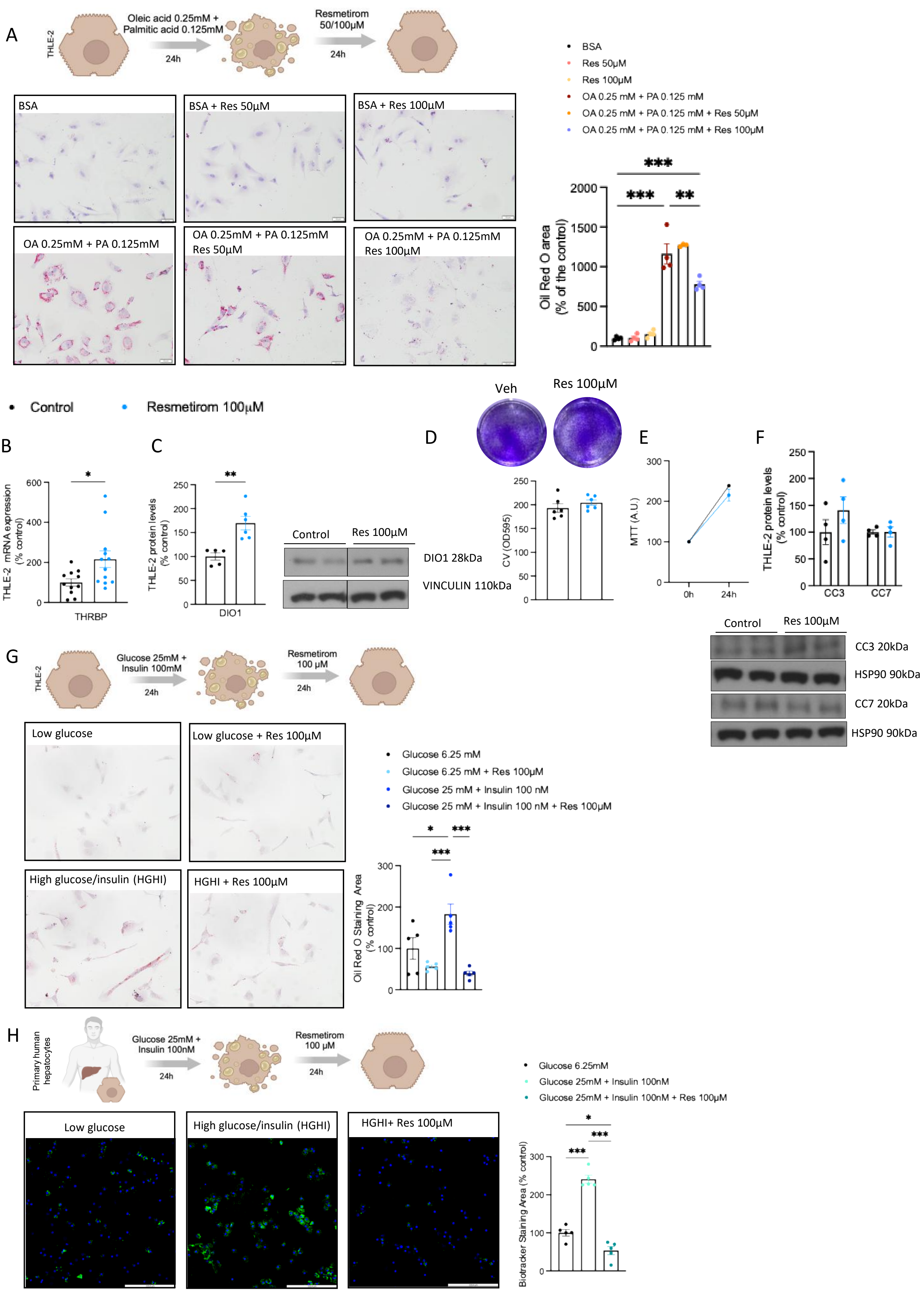

**Supplementary Figure 2. Resmetirom reduces lipid accumulation in human hepatocytes.** (A) Oil Red O staining in THLE-2 cells treated with oleic acid (OA, 0.25 mM) and palmitic acid (PA, 0.125 mM) for 48h in the presence or absence of Resmetirom (50, 100 µM, 24h) (n=4). (B) Relative mRNA of THRBP and (C) DIO1 protein levels in THLE-2 cells treated with Resmetirom compared to control (100 µM, 24 h) (n=5-6). (D) CV staining in THLE-2 cells treated with Resmetirom compared to control (100 µM, 24 h) (n=6). (E) MTT assay in THLE-2 cells treated with Resmetirom compared to control (100 µM, 24 h) (n=6). (F) Western blot analysis of cleaved caspase-3 and cleaved caspase-7 protein levels in THLE-2 cells treated with Resmetirom (100 µM, 24 h) compared to control (n=4). (G) Oil Red O staining in THLE2- cells incubated with high glucose and high insulin, with or without Resmetirom treatment (100 µM, 24 h) (n=5). (H) Biotracker staining in primary human hepatocytes incubated with high glucose and high insulin, with or without Resmetirom treatment (100 µM, 24 h) (n=5). Data are presented as mean ± SEM. Statistical significance was determined using a t-test or one-way ANOVA followed by Bonferroni's multiple comparison test, with significance levels indicated as follows: *$p < 0.05$, **$p < 0.01$, ***$p < 0.001$.

## Supplementary Figure 3

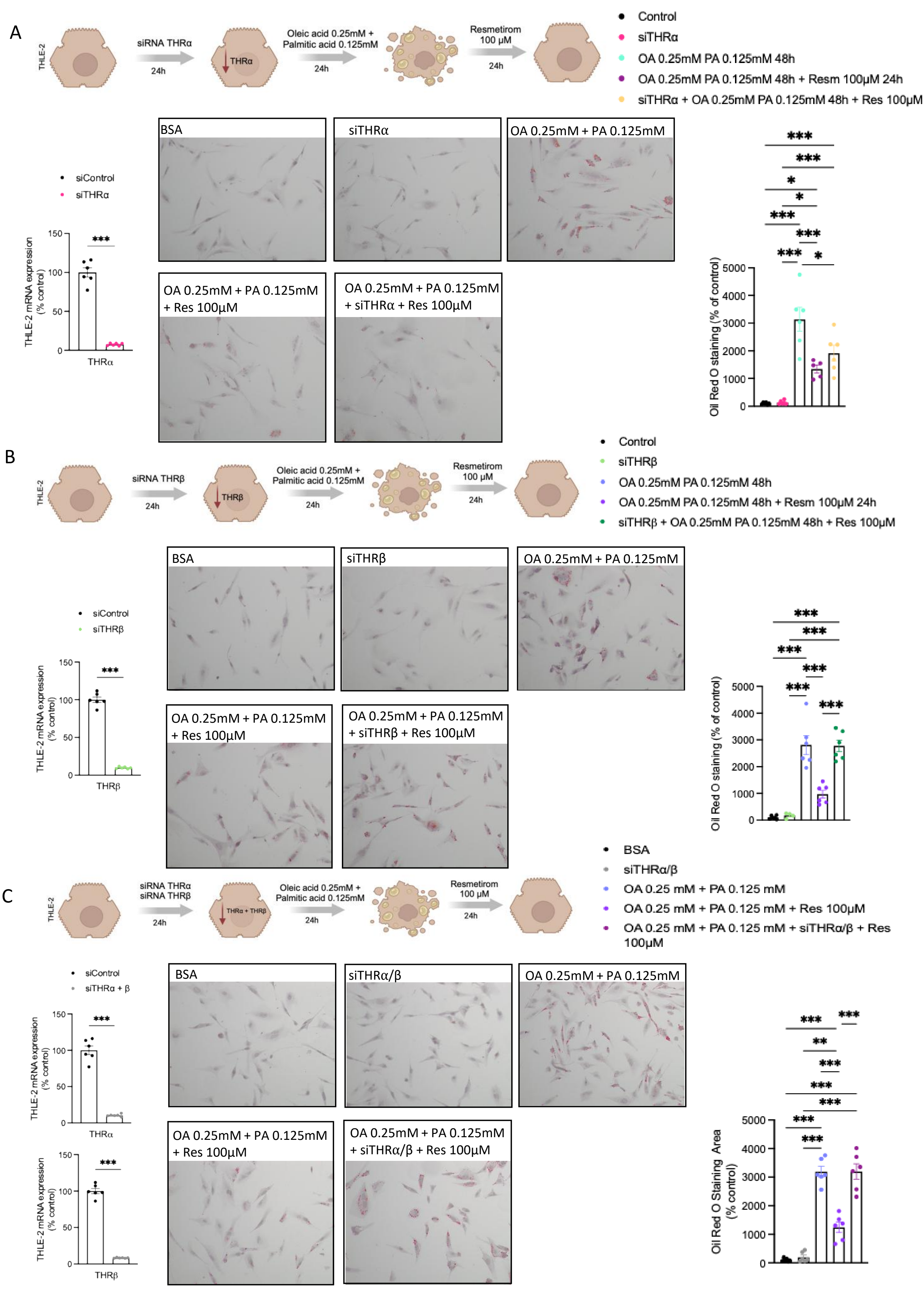

**Suplemmentary Figure 3. Resmetirom acts in human hepatocytes through its selective action on the thyroid hormone receptor beta.** (A) Oil Red O staining in THLE-2 cells, with or without THR$\alpha$ expression, following treatment with oleic acid (OA, 0.25 mM) and palmitate (PA, 0.125 mM) for 12 hours, and subsequent exposure to Resmetirom (100 µM) for another 24 hours (n = 6). (B) Oil Red O staining in THLE-2 cells, with or without THRβ expression, subjected to the same treatment conditions as described in (A) (n = 6). (C) Oil Red O staining in THLE-2 cells, with or without THR$\alpha$ and THRβ expression, following identical treatment conditions (n = 6). Data are presented as mean ± SEM. Statistical significance was determined using a t-test or a one-way ANOVA followed by Bonferroni's multiple comparison test, with significance levels indicated as follows: *$p < 0.05$, **$p < 0.01$, ***$p < 0.001$.

# Supplementary Figure 4

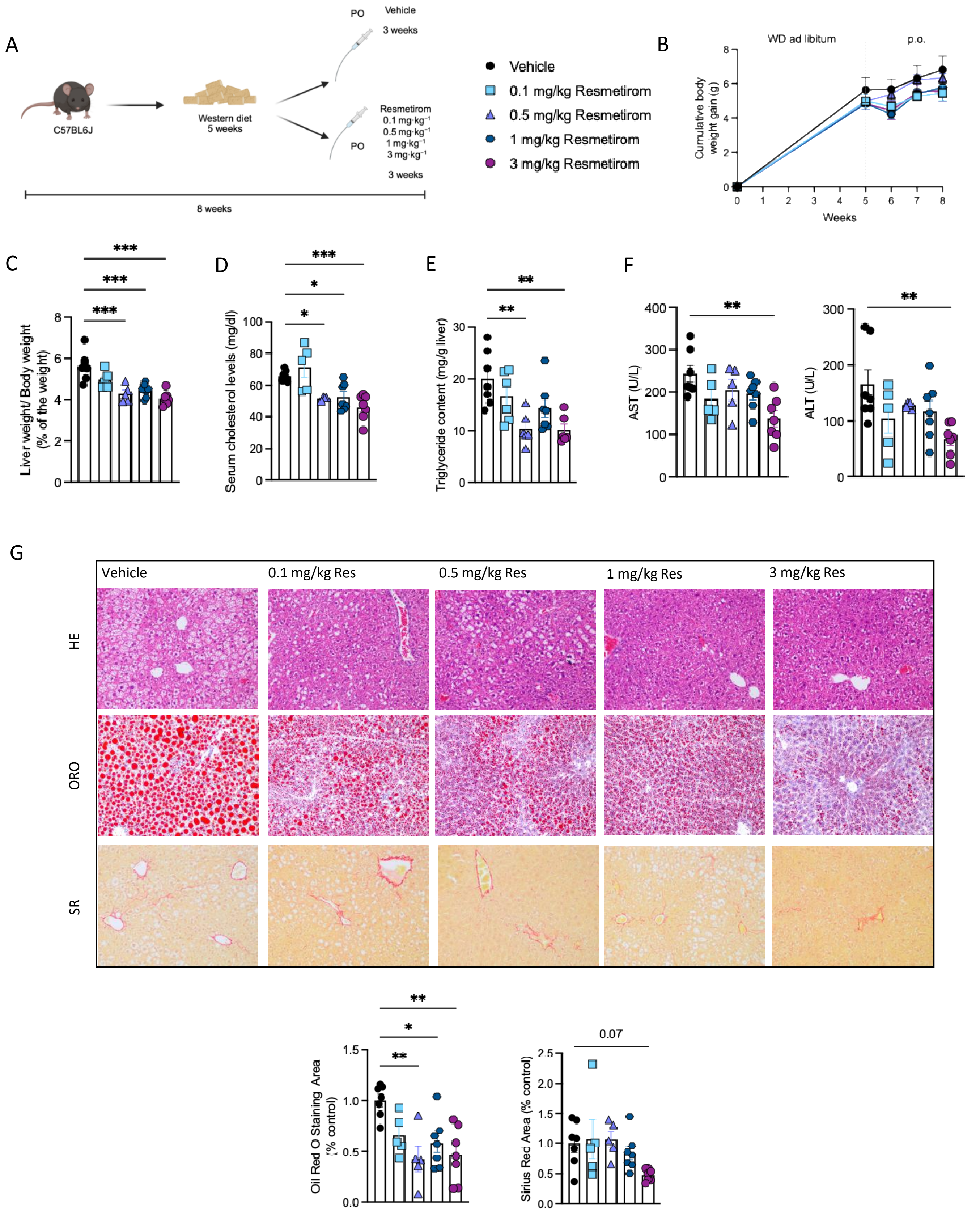

**Suplemmentary Figure 4. Dose response *in vivo* of Resmetirom indicates 3mg/kg is capable to ameliorate MASH in mice fed Western-diet during 8 weeks.** (A) Mice were fed a Western Diet (WD) for five weeks to induce MASLD/MASH, followed by a daily oral treatment with either a vehicle or Resmetirom for three weeks (n= 5-7 per group). (B) Cumulative body weight gain. (C) Liver weight in mice treated orally with different doses of Resmetirom (0.1 mg/kg, 0.5 mg/kg, 1 mg/kg and 3 mg/kg) for 3 weeks. (D) Serum cholesterol levels and (E) triglyceride content levels. (F) Serum levels of AST and ALT. (G) Representative micrographs of Oil Red O staining (lipid accumulation) and Sirius Red staining (collagen deposition) in liver sections. Data are presented as mean ± SEM. Statistical significance was determined by a one-way ANOVA followed by Bonferroni's multiple comparison test, with significance levels indicated as follows: $*p < 0.05$, $**p < 0.01$, $***p < 0.001$.

## Supplementary Figure 5

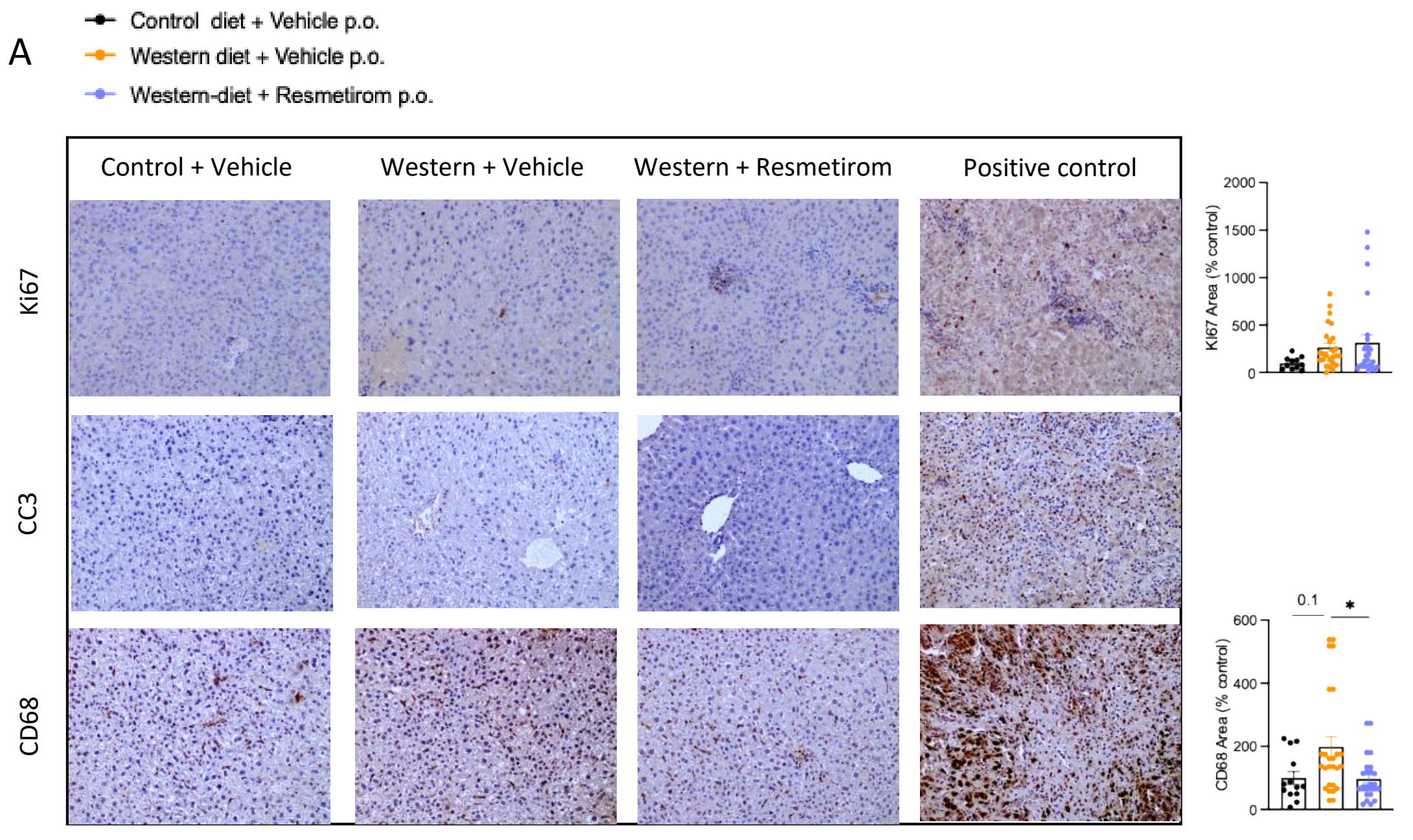


**Supplementary Figure 5. Resmetirom does not affect proliferation or apoptosis and reduces inflammation.** (A) Representative micrographs of Ki67 (proliferation), Cleaved-Caspase 3 (cell death) and CD68 (inflammation) in liver sections of mice fed control diet, Western-diet and Western-diet and Resmetirom p.o. Statistical significance was determined using a one-way ANOVA followed by Bonferroni's multiple comparison test, with significance levels indicated as follows: *$p < 0.05$.

**Supplementary Figure 6**

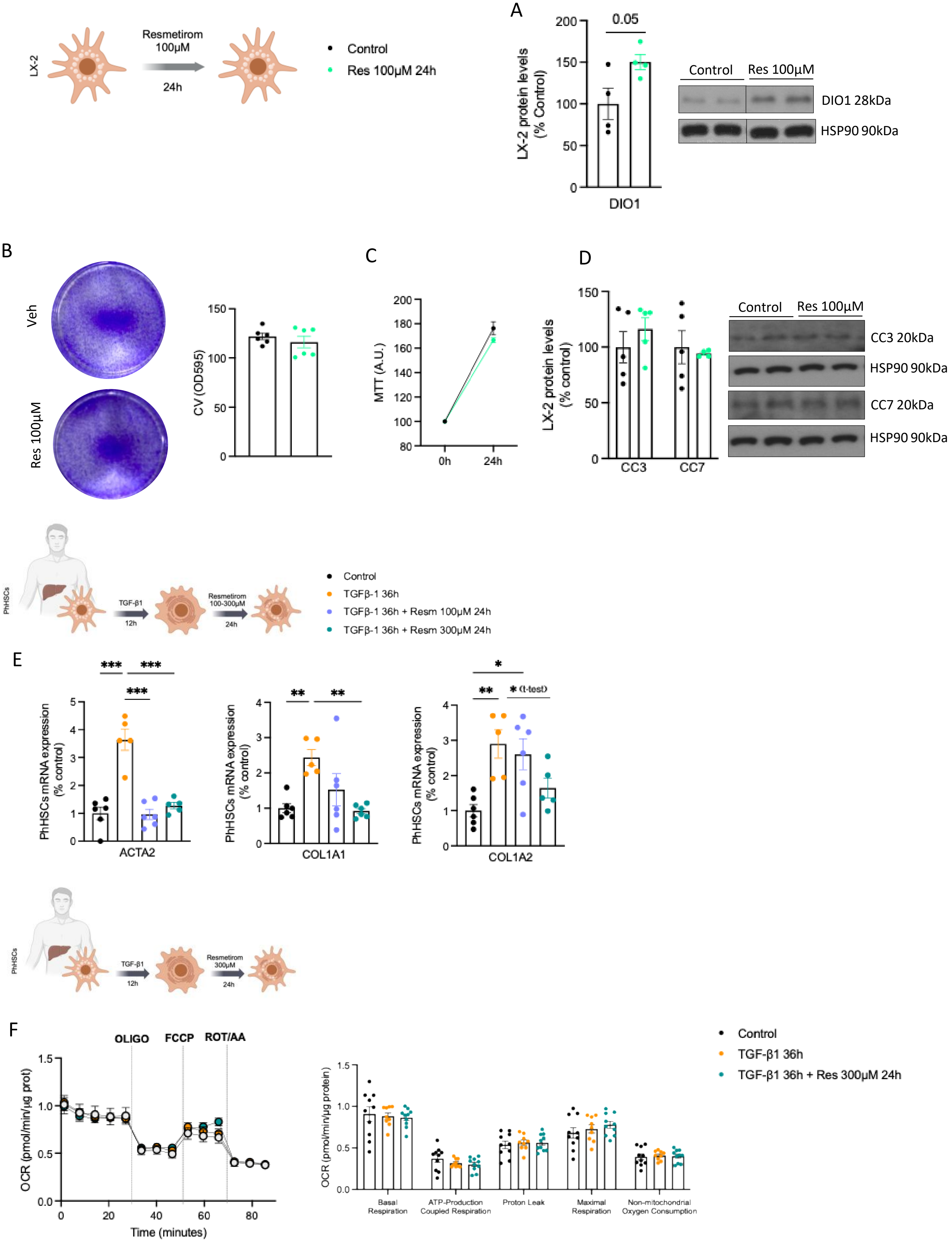

**Supplementary Figure 6. Effects of Resmetirom on migration and cell viability in human hepatic stellate cells (LX-2).** (A) DIO1 protein levels in lx-2 cells treated with Resmetirom compared to control (100 µM, 24 h) (n=4). (B) CV staining in LX-2 cells treated with Resmetirom compared to control (100 µM, 24 h) (n=6). (C) MTT assay in LX-2 cells treated with Resmetirom compared to control (100 µM, 24 h) (n=6). (D) Western blot analysis of cleaved caspase-3 and cleaved caspase-7 protein levels in LX-2 cells treated with Resmetirom (100 µM, 24 h) compared to control (n=5). (E) Fibrotic markers in PhHSCs treated with TGF-β1 during 36h, followed by treatment with either a vehicle or Resmetirom (100-300 µM) for 24h (n= 5-6 per group). (F) OCR measurement in PhHSCs treated with TGF-β1 during 36h, followed by treatment with either a vehicle or Resmetirom (300 µM) for 24h (n= 9-10 per group). Data are presented as mean ± SEM. Statistical significance was determined using a t-test or one-way ANOVA followed by Bonferroni's multiple comparison test.

**Supplementary Figure 7**

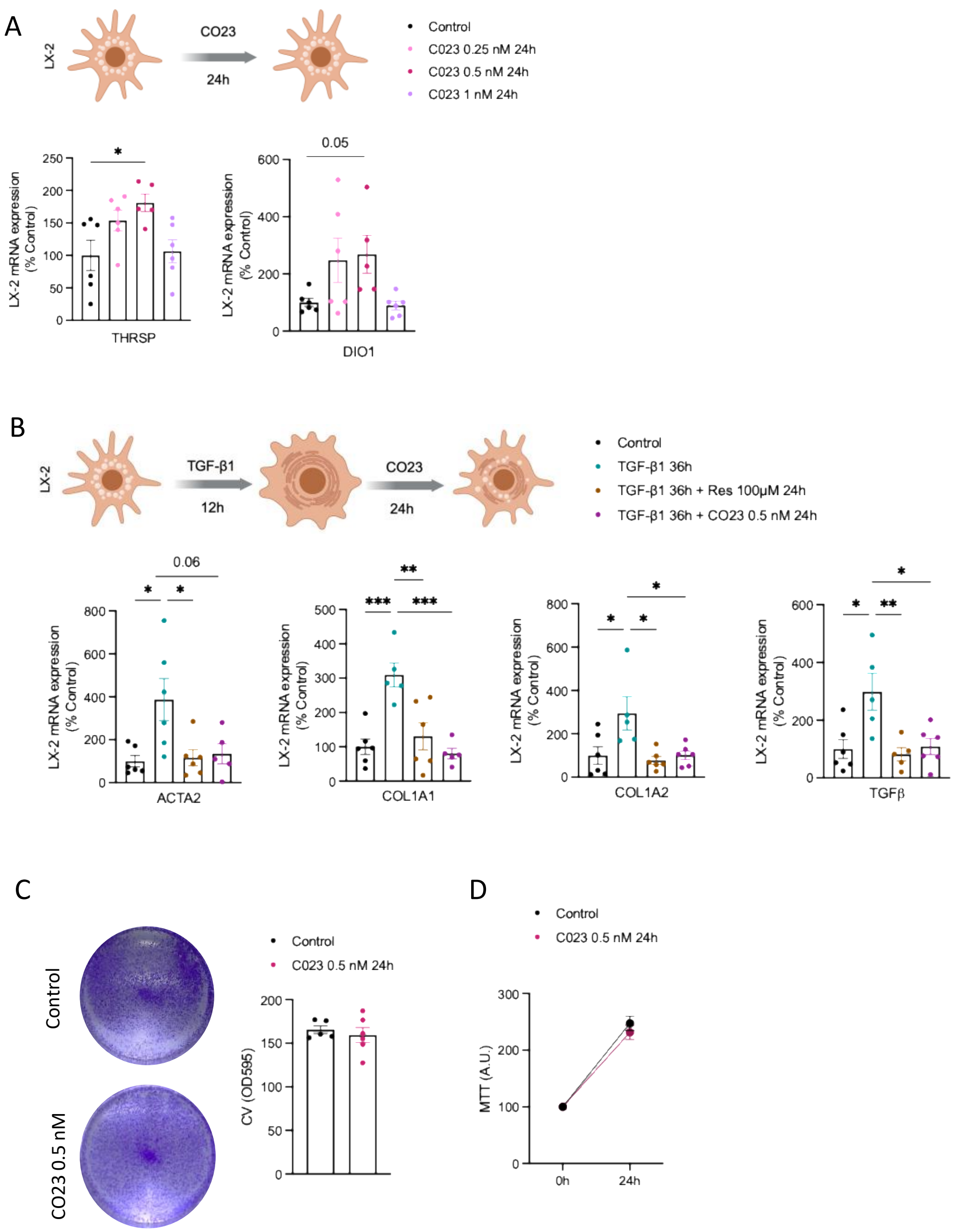


**Supplementary Figure 7. Agonist of THR*α* CO-23 reduces fibrosis markers in active HSCs.** (A) mRNA expression of THRSP and DIO1 in LX-2 cells, treated with a dose response of CO-23 (0.25nM, 0.5nM, 1nM) during 24h (n=5-6). (B) mRNA of liver fibrosis markers in LX-2 cells treated with TGF-β1 (12h) and subsequently with Resmetirom (100µM) or CO-23 (0.5nM) during 24h (n=5-6). (C) Violet crystal and (D) MTT assay of LX-2 cells treated with or without CO-23 (0.5nM) during 24h (n=5). Data are presented as mean ± SEM. Statistical significance was determined using a t-test or one-way ANOVA followed by Bonferroni's multiple comparison test.

**Supplementary Table 1.** Macronutrients composition of rodent diets used.

| Rodent Diets | Chow Diet (Ssniff, TD.88137) | | Western Diet (Ssnif, CD.88137) | |
|---|---|---|---|---|
| | gm % | kcal % | gm % | kcal % |
| **Protein** | 17.5 | 18 | 17.3 | 15 |
| **Carbohydrates** | 64.7 | 69 | 48.7 | 43 |
| **Fat** | 5.1 | 13 | 21.1 | 42 |
| **Total** | | 100 | | |
| **Kcal/gm** | 3.75 | | 4.575 | 100 |

**Supplementary Table 2.** Primers and Taqman probes used.

| Primer | Forward 5'-3' | Reverse 3'-5' |
|---|---|---|
| ***Hs GAPDH*** | GTCTCCTCTGACTTCAACAGC | ACCACCCTGTTGCTGTAGCCAA |
| ***Hs FAAH*** | CTGGAGACCAAACAGAGCCTTG | CACTGAAGAGCCCACCTGTTGA |
| ***Hs THRSP*** | CATTGGCTAGTGAACTGCTCC | AAATGACGGGACAAGTTTGG |
| ***Hs TGF$\beta$*** | GGACCAGTGGGGAACACTAC | AGAGTCCCTGCATCTCAGAGT |
| ***Hs ACTA2*** | TTCAATGTCCCAGCCATGTA | GAAGGAATAGCCACGCTCAG |
| ***Hs SLCO1B1*** | CGTAGAGCAACAGTATGGTCAGC | TTGGCAATTCCAACGGTGTTCAG |
| ***Hs SLCO1B3*** | GTCACCTTGTCTAGCAGGATGC | GCATTCACCCAAGTGTGCTGAG |
| ***Mm Gapdh*** | CATCACTGCCACCCAGAAGACT | ATGCCAGTGAGCTTCCCGTTCAG |
| ***Mm Faah*** | TGTTCCGCTTGGACTCCACCAT | GACTCTGCTTGGTCTCCATCAC |
| ***Mm Col1a1*** | CCTAATGCTGCCTTTTCTGC | ATGTCCCAGCAGGATTTGAG |
| ***Mm Col1a2*** | CCGTGCTTCTCAGAACATCA | CTTGCCCCATTCATTTGTCT |
| ***Mm Acta2*** | CTGACAGAGGCACCACTGAA | CATCTCCAGAGTCCAGCACA |
| | | |
| **Probe** | ID | - |
| ***Hs COL1A1*** | Hs00164004_m1 | |
| ***Hs COL1A2*** | Hs01028956_m1 | |
| ***Hs HPRT1*** | Hs02800695_m1 | |
| ***Hs THRA*** | Hs00268470_m1 | |
| ***Hs THRB*** | Hs00230861_m1 | |
| ***Mm Cpt1a*** | Mm01231183_m1 | |
| ***Mm Thra*** | Mm00579691_m1 | |
| ***Mm Thrb*** | Mm00437044_m1 | |
| ***Mm Hprt1*** | Mm03024075_m1 | |

# Uncropped blots

## Figure 1G.

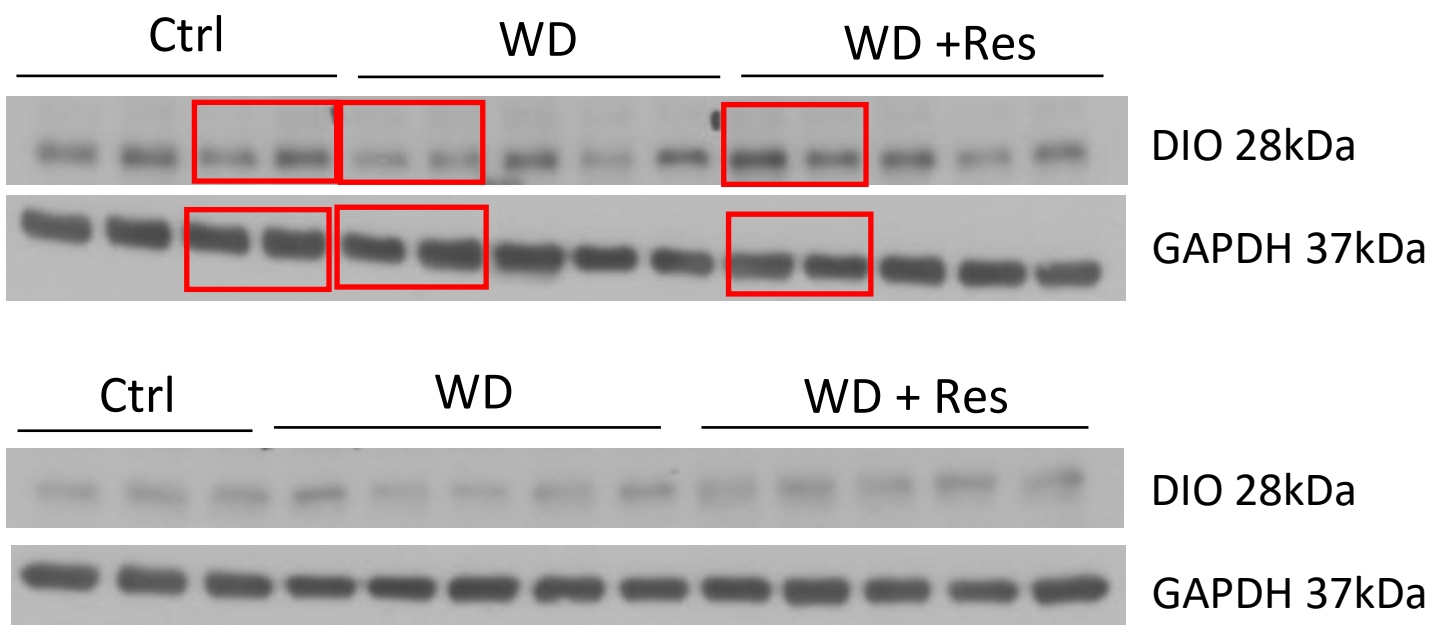


## Supplementary Figure 6D.

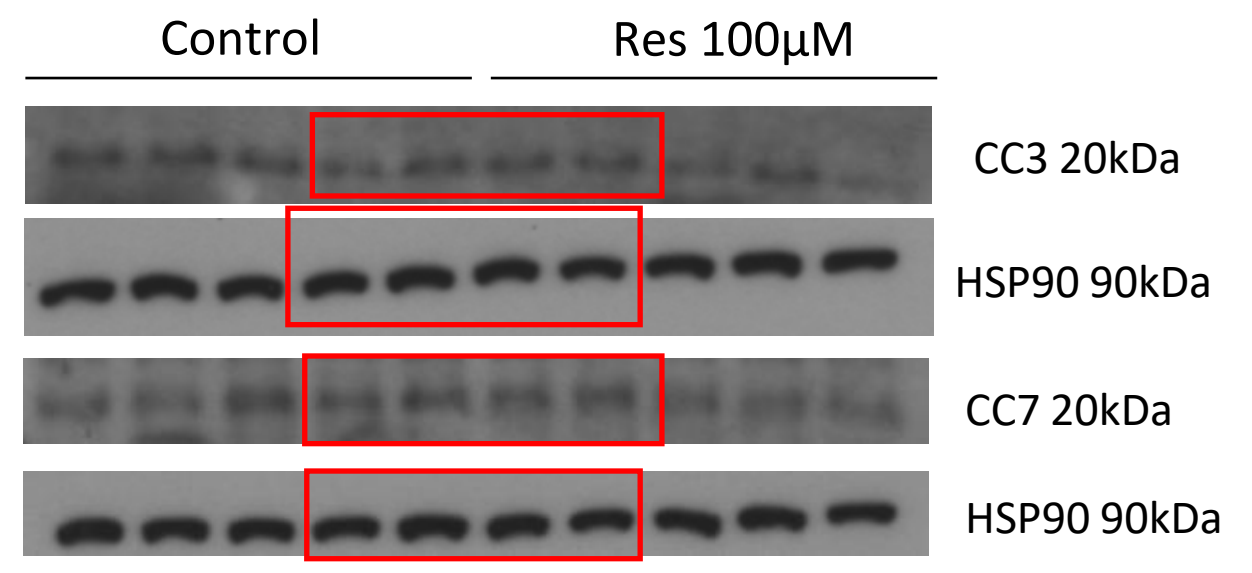


## Figure 2I.

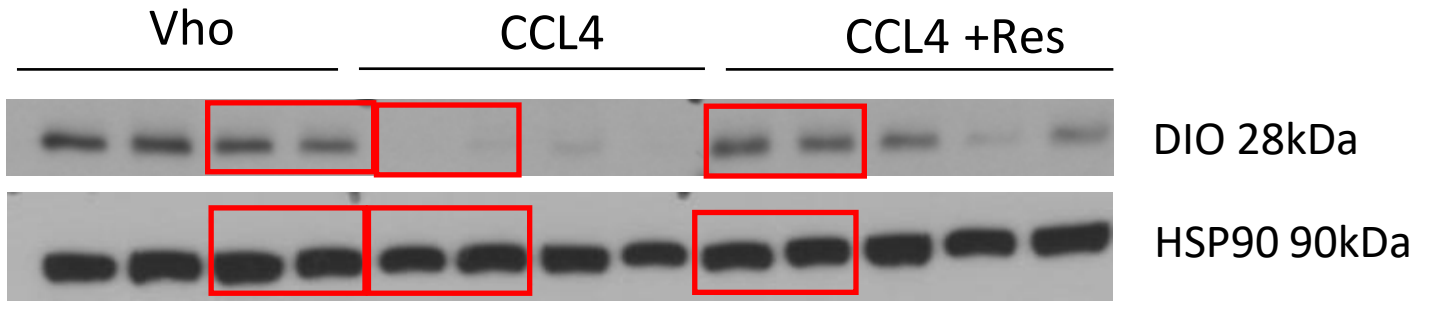


## Supplementary Figure 2C.

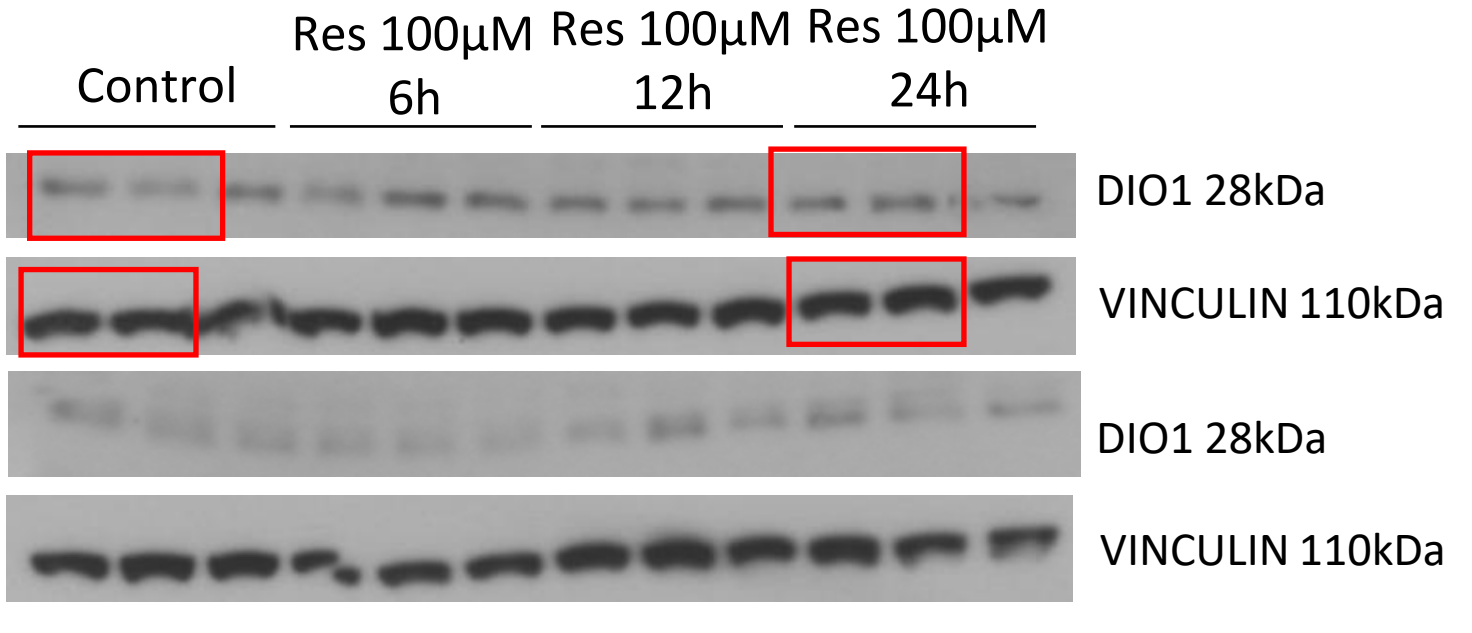


## Supplementary Figure 2E.

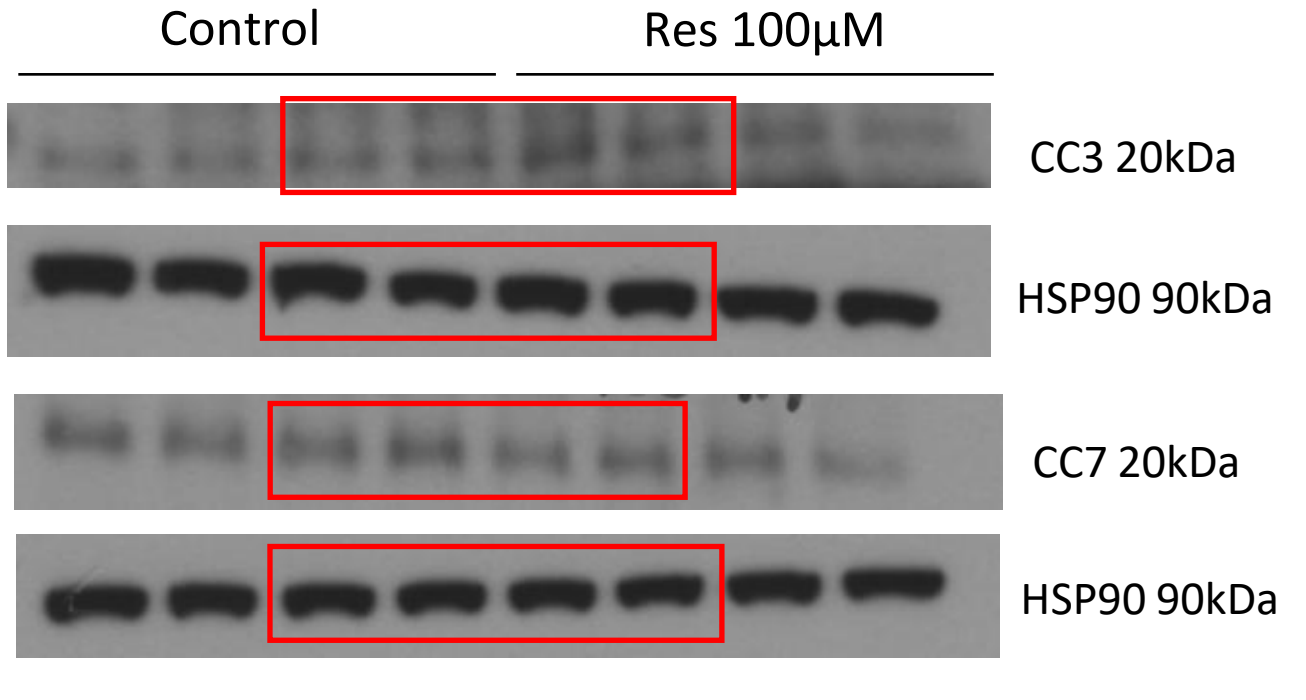


## Supplementary Figure 6A.

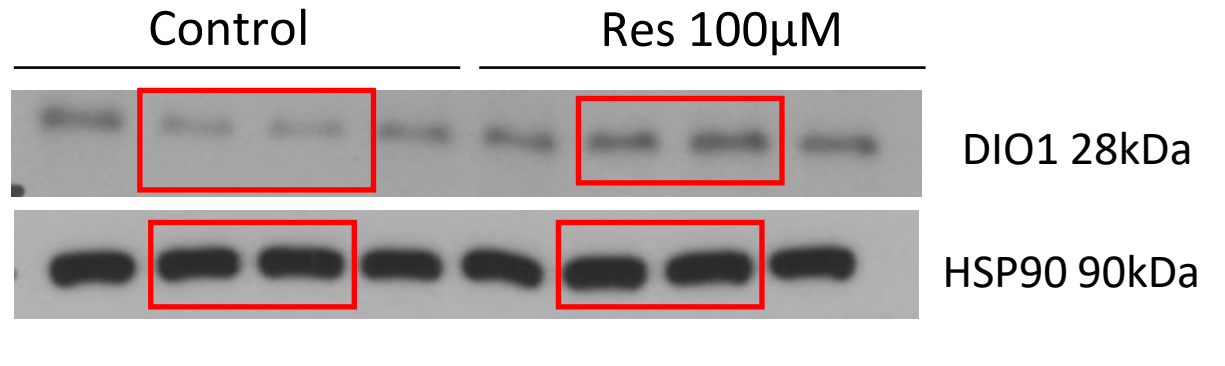

## Methods

*Single nucleus RNA-seq dataset analysis*

Human Single-Nucleus RNA sequencing data from healthy and cirrhotic areas from MASLD livers were obtained from GEO accessions: GSE185477 and GSE174748. The Seurat package (v4.3.0) in R was used for subsequent analysis of the 10X CellRanger count outputs obtained from Gene Expression Omnibus. For quality control, nuclei with mitochondrial content > 30% were removed. Principal component analysis was performed using the top 3,000 most variable genes and UMAP analysis was performed with the top 20 PCAs. Clustering was performed using a resolution of 0.5. For cell annotation we use the Azimuth Human Liver Reference (https://azimuth.hubmapconsortium.org/references/#Human%20-%20Liver).

*Cell line cultures*

The THLE-2 human hepatic cell line (CRL-2706, Lot No: 70026207, ATCC) was cultured in bronchial epithelial cell basal medium (BEBM), supplemented with a growth factor BulletKit (Lonza/Clonetics Corporation), 70 ng/mL phosphoethanolamine, 5 ng/mL epidermal growth factor, 10% (v/v) fetal bovine serum (FBS), and 1% (v/v) Glutamine-Penicillin-Streptomycin solution (MERCK). THLE-2 cells were grown on culture plates pre-coated with a mixture of 0.01 mg/mL fibronectin (#33010018, Sigma Aldrich), 0.01 mg/mL bovine serum albumin (#A4503, Sigma Aldrich), and 0.03 mg/mL type I collagen (#sc-136157, Santa Cruz).

The LX-2 human hepatic stellate cell line (CL-0560, Lot No: 8501J141804, Elabscience) was cultured in Dulbecco´s Modified Eagle´s Medium (DMEM) with high glucose (D5796, MERCK), supplemented with 2% (v/v) FBS and 1% (v/v) Penicillin-Streptomycin solution. Both cell lines were maintained at 37° C in a humid atmosphere containing 5% CO2.

*Oleic and Palmitic Acid Experiments*

In the experiment involving oleic and palmitic acid, 0.5 x 10^5 THLE-2 cells and human primary hepatocytes were seeded in a 24-well plate. Both control and treated cells were exposed to serum-free medium supplemented with a combination of 0.25 mM oleic acid

(MERCK) and 0.125 mM palmitate (MERCK) bound to fatty acid-free BSA (Capricorn) at a 2:1 molar ratio for 24 hours to induce lipid accumulation [1]. Control cells were supplemented with fatty acid-free BSA alone. After 24 hours of exposure to 0.25 mM oleic acid + 0.125 mM palmitate, the cells were treated with Resmetirom (25, 50 and 100 μM) for 24 hours. Cells were subsequently stained with Oil Red O to visualize lipid accumulation.

*TGF-β1 treatment*

LX-2 cell line, and primary HSCs were incubated with medium supplemented with recombinant human TGF-β1 (100-21, PreproTech) at a concentration of 8 ng/mL or vehicle for 36 h.

*Resmetirom treatment*

Resmetirom (MGL-3196, MedChemExpress) was dissolved in dimethyl sulfoxide (DMSO, D2653-5x10ml, Merck) according to the manufacturer's instructions. For the *in vivo* experiments, Resmetirom was administered once daily in the morning via oral gavage at a dose of 0.1, 0.5, 1, and 3 mg/kg. The control groups received the vehicle, DMSO, at an equivalent dose. This procedure was carried out over a period of three weeks.

For the *in vitro* experiments, a concentration-response analysis of Resmetirom was performed for each cell type utilized. Based on these results, the final concentrations selected were 25, 50, and 100 μM for THLE-2, 50 and 100 μM for LX-2, 300 μM for PhHSCs, and 500 μM for PmHSCs during 24h.

*Crystal Violet assay*

0.5 x 10^5 cells per well in 24-well plates and allowed to adhere overnight. After treatment with Resmetirom and CO23 for 24 hours, cells were washed with PBS, fixed with 100 μL of 4% paraformaldehyde for 15 minutes, and washed again with PBS. The cells were then stained with 100 μL of 0.5% crystal violet solution for 15 minutes at room temperature, followed by several washes with water to remove excess dye. After drying,

crystal violet was solubilized with 500 µL of methanol to each well for 10-15 minutes, and absorbance was measured at 590 nm using a microplate reader.

*MTT assay*

Cells were seeded at a density of 1 x 10^4 cells per well in 96-well plates and allowed to adhere overnight. After treatment with Resmetirom and CO23 for 24 hours, 10 µL of MTT solution (5 mg/mL in PBS) was added to each well and cells were incubated for 1 hour at 37ºC. The medium was then removed, and 100 µL of DMSO was added to solubilize the formazan crystals. The absorbance was measured at 570 nm using a microplate reader.

*Cell Transfections*

THLE-2, LX-2 and phHSCs cells were transfected with specific small interfering RNAs (siRNAs) to silence the expression of THRα (siGENOME SMARTPool, L-003446-00-0005, Dharmacon) and THRβ (siGENOME SMARTPool, L-003447-00-0005, Dharmacon). In addition, FAAH (siGENOME SMARTPool, L-009907-00-0005, Dharmacon) was silenced in LX-2 cells. Non-targeting siRNAs (D-001810-10-05 or D-001206-13-05, Dharmacon) were used as negative controls.

THLE-2 cells were cultured 24 hours before transfection. Transfections were performed using Dharmafect 1 reagent (T-2001-03, Dharmacon), following the manufacturer's protocol. Briefly, 50 pmol of each siRNA was diluted in 200 µL of OptiMEM (#31985-070, Life Technologies) and mixed with 6.5 µL of Dharmafect 1, pre-diluted in 193.5 µL of OptiMEM. The final mixture (500 µL) was added to each well. After six hours, the transfection medium was replaced with fresh BEGM, and the corresponding treatments were applied.

LX-2 and phHSCs cells were cultured for 24 hours before transfection. Transfections were performed using Lipofectamine RNAiMAX (13778100, Invitrogen). For each well, 0.05 nmol of siRNA was diluted in 250 µL of OptiMEM and mixed with 5 µL of Lipofectamine RNAiMAX, pre-diluted in 245 µL of OptiMEM. The resulting 500 µL mixture was added directly to the cells. After twenty-four hours, the transfection medium was replaced with fresh medium.

To induce SLCO1ß1 overexpression in THLE-2 and LX-2 cells, we transfected them with a plasmid encoding the SLCO1ß1 sequence (Vector Builder, #VB250513-1373ekh). An empty plasmid was used as a negative control. Transfection was carried out using Lipofectamine 2000 (Invitrogen, #11668-019). Briefly, 0.5 ng (THLE-2) or 15 pg (LX-2) of plasmid DNA were diluted in 250 µl of Opti-MEM (Life Technologies, #31985070) and combined with 6 µl of Lipofectamine 2000 previously diluted in 250 µl of Opti-MEM. The resulting complex was added to each well along with 500 µl of fresh medium and incubated overnight. The following day, the transfection mixture was replaced with fresh culture medium.

*Human Primary Hepatocytes and Human Primary Hepatic Stellate Cells*

Human primary hepatocytes (NHM2354B-HE-N, BeCytes Biotechnologies) were thawed using Hepatocyte Thawing Medium (MHT-23-14, BeCytes Biotechnologies) in accordance with the manufacturer's instructions. Following centrifugation, the cells were resuspended and seeded in Hepatocyte Plating Medium (HPM-250, BeCytes Biotechnologies). After a six-hour incubation period, the medium was replaced with Hepatocyte Maintenance Medium (MHM-250, BeCytes Biotechnologies), supplemented as per the specific treatment requirements. Primary hepatocytes were maintained at 37°C in a humidified atmosphere containing 5% $CO_2$.

Human primary hepatic stellate cells (CyHum19007-SC-P2-Z, BeCytes Biotechnologies) were thawed using Thawing Medium (MHT-NPC50, BeCytes Biotechnologies) following the manufacturer's guidelines. Subsequently, the cells were seeded and cultured in Growth Medium for Stellate Cells (HMSG-50, BeCytes Biotechnologies). These primary cells were maintained at 37°C in a humidified atmosphere containing 5% $CO_2$.

*Hyperglycemia-Hyperinsulinemia conditioned medium*

THLE-2 cells were seeded at a density of 0.5 x 10^5 cells per well in a 24-well plate for Oil Red O staining. To investigate lipogenesis *in vitro*, the cells were cultured in Krebs-Henseleit-HEPES (KHH; 120 mmol/L NaCl, 4.7 mmol/L KCl, 2.5 mmol/L $CaCl_2$, 1.2 mmol/L $MgSO_4$, 1.2 mmol/L $KH_2PO_4$, 25 mmol/L $NaHCO_3$, 25 mmol/L HEPES, pH 7.4) with either low (6.25 mM) or high glucose concentration (25 mM) (D-glucose,

#G8270, Sigma-Aldrich) and high insulin (100 nM) (Actrapid, Novo Nordisk), with or without Resmetirom (100 μM) for 24 hours.

Primary human hepatocytes (NHM2354B-HE-N, BeCytes Biotechnologies) were seeded at a density of 0.4 x 10^5 cells per well in a 24-well plate for Biotracker staining (BioTracker 488 Green Lipid Dye, Cat. #SCT144, Merck) using Hepatocyte Plating Medium (HPM-250, BeCytes Biotechnologies). After 6 hours, the cells were transitioned to Hepatocyte Maintenance Medium (MHM-250, BeCytes Biotechnologies), supplemented with up to 25 mM glucose (D-glucose, #G8270, Sigma-Aldrich) and 100 μM insulin (Actrapid, Novo Nordisk). Cells were then treated with or without Resmetirom (100 μM) for 24 hours.

*Biotracker staining*

After fixation with PFA 4% for 15 minutes, cells were washed with PBS and staining with Biotracker (BioTracker 488 Green Lipid Dye, Cat. #SCT144, Merck) in a humid chamber during 15 minutes at RT. Then, primary human hepatocytes were mounted with a fluorescence-compatible medium (Fluoro-Gel with Tris buffer, Electron Microscopy Science, Cat #17985-10) with DAPI 1:1000 (D9542, Sigma-Aldrich) and subjected to microscopic examination. Five representative microphotographs of each sample were taken with a Thunder Imager tissue microscope (Leica Microsystems). Leica Las X 3.7.4 software was used to acquire and analyze immunofluorescence staining. Image J 1.52p software was used for the quantification of the staining area.

*Serum levels of metabolites*

During the sacrifice of mice, whole trunk blood was collected and then spun for 10 min at 6000g and 4°C. The supernatant was transferred to a new tube to obtain the serum. Serum alanine aminotransferase (ALT, 41283, Spinreact), aspartate aminotransferase (AST, 41273, Spinreact) activities and serum cholesterol levels (1001090, Spinreact) were measured by spectrophotometry using a ThermoScientific Multiskan GO spectrophotometer, following the manufacturer's instructions. Briefly, 3 μL of serum was pipetted into the wells of a microplate, followed by the addition of the specific reagents. The reaction mixture was incubated at 37°C for the recommended time (typically 10-15 minutes). Absorbance was measured at 505 nm using a microplate reader or

spectrophotometer. Cholesterol levels were calculated from a standard curve generated from the cholesterol standards provided with the kit. Results were expressed as mg of cholesterol per deciliter of serum.

*Triglyceride content*

Liver tissue samples were homogenized in phosphate-buffered saline (PBS), and triglycerides were extracted using a chloroform:methanol (2:1) solution following the Bligh and Dyer method. After phase separation, the lower organic phase containing the lipids was collected, evaporated, and re-suspended in chloroform. Triglyceride content was measured using the Spinreact Triglyceride Assay Kit (100130, Spinreact), according to the manufacturer´s instructions. Briefly, 10 μL of the lipid extract was added to the microplate wells, followed by the addition of assay reagent. The reaction mixture was incubated at 37° C for 5 minutes, and the absorbance was measured at 500-550 nm using a microplate reader. Triglyceride concentrations were determined by comparing the absorbance values to a standard curve generated from the triglyceride standards provided in the kit. Results were expressed as mg of triglyceride per g of liver tissue.

*Histological procedures*

<u>*Oil Red O staining.*</u> Frozen tissue was sectioned at 10 μm thickness using a cryostat. Sections were mounted on slides and fixed in formalin overnight. The following day, samples were washed under running tap water for several minutes. Sections were then incubated in the working Oil Red O solution (Sigma-Aldrich; 00625) for 20 minutes, protected from light, followed by washing under running tap water for 20 minutes. Subsequently, sections were counterstained with Harris hematoxylin (Bio-Optica; 05-06004/L) for 5 minutes and rinsed under running tap water for 5 minutes to allow bluing. Finally, the sections were mounted using an aqueous mounting medium (Bio-Optica; 05-1740).

Cells were seeded on sterile glass coverslips placed in the wells of a 24-well plate. Following treatment, the culture medium was removed, and cells were gently rinsed with phosphate-buffered saline (PBS). Fixation was carried out using 10% neutral buffered formalin for 10 minutes at room temperature. After fixation, wells were washed with PBS, and 200 μL of freshly prepared working Oil Red O solution was added to each well. Cells

were incubated for 20 minutes at room temperature, followed by thorough washing with distilled water until the background was clear. Subsequently, a counterstaining step was performed by incubating the coverslips with Mayer's haematoxylin for 10 minutes, after which they were rinsed again with distilled water. Coverslips were then carefully removed and mounted cell-side down onto glass microscope slides using an aqueous mounting medium. Stained preparations were examined under a light microscope to evaluate intracellular lipid droplet accumulation.

*Hematoxylin and Eosin staining*. Liver samples were fixed in 4% formaldehyde for 24 hours, dehydrated through increasing concentrations of ethanol, cleared in xylene, and embedded in paraffin. Sections of 4 µm thickness were cut, deparaffinized in xylene, and rehydrated through decreasing concentrations of ethanol to distilled water. Sections were stained with Harris hematoxylin (BioOptica; 05-06004/L) for 10 minutes and rinsed in running tap water for 10 minutes. Subsequently, sections were counterstained with Eosin (Bio-Optica; 05-100009E) for 5 minutes according to the manufacturer's instructions. After staining, sections were dehydrated through graded ethanol, cleared in xylene, and mounted with a permanent mounting medium DPX (Sigma-Aldrich; 1.00579). Slides were then left to dry overnight in a fume hood.

*Sirius Red staining.* Paraffin-embedded samples were dewaxed and rehydrated through graded alcohols. Sections were first stained with Weigert´s hematoxylin (Bio-Optica; 05-B06008) for 10 minutes and rinsed under running tap water for 10 minutes to allow proper bluing. Subsequently, the samples were stained with PicroSirius Red solution for 1.5 hours. After staining, sections were dehydrated through graded alcohols, cleared in xylene, and finally mounted using a permanent mounting medium DPX (Sigma-Aldrich; 1.00579). Slides were then left to dry overnight in a fume hood.

*Immunohistochemistry.* Paraffin-embedded tissue sections were dewaxed, hydrated, and subjected to antigen retrieval in PT Link (DAKO) using either TE buffer pH 9 (for Ki67, Abcam ab16667, 1:500, and $\alpha$-SMA, Abcam 124964, 1:2000) or citrate buffer pH 6 (for cleaved caspase-3, Cell Signaling 9661, 1:200, and CD68, Abcam ab125212, 1:1000). Endogenous peroxidase activity was blocked with 3% hydrogen peroxide for 10 min. Sections were then incubated overnight at 4 °C with the primary antibodies. After washing, samples were incubated with the EnVision secondary system (DAKO SM802) for 30 min at room temperature. Bound antibodies were visualized using DAB substrate (DAKO

K346811) for 1 min, and nuclei were counterstained with Mayer's hematoxylin for 10 min. Sections were then dehydrated and mounted.

In these histological staining techniques, up to 3 representative microphotographs of each animal were taken with a BX51 microscope equipped with a DP70 digital camera (Olympus). Lipids in Oil Red O- stained sections, collagen depositions in Sirius Red-stained sections and positive area in CC3 and CD68 immunohistochemistry were quantified using ImageJ 1.52p software.

*Isolation and Culture of Murine Hepatocytes and HSCs*

Primary hepatocytes and HSCs were isolated from murine liver tissue using a multi-step procedure involving Hanks 10X Buffer (NaCl 1.4M, KCl 0.05M, $MgSO_4 \cdot 7H_2O$ 0.01M, $Na_2HPO_4 \cdot 2H_2O$ 3mM, $KH_2PO_4$ 4mM). Initially, the necessary reagents were prepared, beginning with Hanks 10X, which was subsequently used to prepare Hanks Hepes (Hanks 10X, $NaHCO_3$ 25mM, Hepes 12mM), Hanks 1 (Hanks Hepes, EGTA 0.6 mM, BSA 0.23 mM, Sodium Heparin 0.1ml/50ml), and Hanks 2 (Hanks Hepes, $CaCl_2$ 4mM, Collagenase IV) [2, 3]. For perfusion, the animal was anesthetized, and the surgical site was sterilized. The liver was perfused through the portal vein with Hanks 1 and Hanks 2 solutions, followed by in vitro digestion using Hanks 2. The liver samples were then kept at 4ºC, and both cells and liver remnants were filtered and centrifuged at 150g to separate hepatocytes from non-parenchymal cells (NPCs).

To isolate hepatocytes, the resulting pellet was resuspended in 25 mL of cold PBS using a serological pipette, performing a gentle up-and-down motion. The mixture was then diluted to 50 mL to thoroughly cleanse the cells. Once a clean pellet was obtained, the supernatant was discarded, and the pellet was resuspended in 15 mL of DMEM F12 medium (containing 10% FBS, 1% PS, 2nM $L^{-1}$ Glutamine, 1% Amphotericin, 1 µM Dexamethasone, and Insulin). The suspension was then filtered through a handheld filter to remove any remaining connective tissue. The hepatocytes were seeded onto collagen-coated plates and incubated for 24 hours to allow for cell adherence, after which they were collected.

For the isolation of HSCs, the supernatant was centrifuged at 800g for 10 minutes. A gradient preparation was performed using 23% Optiprep/iodixanol solution. To prepare the gradient, 2.5 mL of Optiprep was pipetted into a 15 mL tube, followed by the addition of the

resuspended cell pellet in 2.5 mL of GBSS, resulting in an 11.5% Optiprep mixture. This solution was mixed thoroughly, and 2 mL of GBSS was carefully layered on top. The mixture was centrifuged at 1400g for 21 minutes at 4°C without brake (acceleration 5, brake 0). After centrifugation, the light interface between the white and Optiprep layers, which is enriched in HSCs, was carefully collected. This interface was transferred to a new 15 mL tube, diluted with GBSS to a final volume of 15 mL, and centrifuged again at 800g for 10 minutes at 4°C. The pellet was then resuspended in pre-warmed Iscove's Modified Dulbecco's Medium (containing 10% FBS, 1% PS, 2nM $L^{-1}$ Glutamine, and 1% Amphotericin). The resuspended HSCs were seeded into culture wells and collected after 24 hours.

*$CCl_4$ in vivo treatment*

Animals received intraperitoneal injections of carbon tetrachloride (CCl4) at a dose of 0.6 mL/kg body weight, diluted in corn oil, once per week for 6 consecutive weeks. Prior to administration, animals were weighed to calculate the exact dose. Injections were performed under sterile conditions, preferably using a 25G needle, with care to avoid injury to internal organs. Control animals received an equivalent volume of vehicle (corn oil) on the same schedule. Animals were monitored for signs of distress or toxicity throughout the study. At the end of the treatment period, animals were euthanized for tissue collection and further analysis.

*Collagen content assay*

Collagen content was determined using a fluorometric assay kit (Sigma-Aldrich; MAK322), following the manufacturer's instructions. Approximately 10 mg of liver tissue was homogenized in 100 µL of distilled water and transferred to a pressure-tight polypropylene vial. Samples were then mechanically digested to release soluble collagen peptides. For the assay, 20 µL of each sample or collagen standard were loaded into black 96-well plates suitable for fluorescence measurements. Then, 30 µL of the Master Reaction Mix were added to each well and incubated for 60 minutes at 37 °C. Subsequently, 40 µL of the Dye Reagent were added, followed by incubation for 10 minutes at 37 °C. Finally, 8 µL of the Developer solution were added and incubated for an additional 10 minutes at 37 °C. Fluorescence was measured using a microplate reader at excitation/emission wavelengths of

375/465 nm. Collagen content was calculated from the standard curve and expressed as fluorescence (arbitrary units) per mg of tissue.

*Cellular uptake assay of Resmetirom in LX-2*

LX-2 cells (0.4 × 10^6) were seeded in 60 mm culture plates (Corning, 430166). After overnight attachment, the culture medium was replaced with fresh medium supplemented with Resmetirom (100 μM), and cells were incubated for 2 h at either 37°C or 4°C. Following treatment, the medium was removed and cells were washed with cold PBS. Subsequently, 1.5 mL of methanol (MeOH) was added to each plate, and samples were incubated overnight at 4°C. After incubation, samples were centrifuged at 12,000 × g for 15 min at 4°C. The resulting supernatant was collected, evaporated to dryness, and reconstituted in 200 μL of ACN/H2O containing 0.1% formic acid prior to UPLC analysis.

In parallel, control experiments were conducted to evaluate solvent extraction efficiency, non-specific binding to culture plates, and non-specific binding to cellular membranes. For solvent extraction efficiency, LX-2 cells were treated with vehicle (DMSO) at 37°C for 2 h. To assess non-specific binding to culture plates, experiments were performed in empty plates without cells, which were incubated with 100 μM Resmetirom for 2 h. Non-specific binding to cellular membranes was evaluated by incubating LX-2 cells with 100 μM Resmetirom at 4°C for 2 h to minimize active cellular uptake.

All samples were analyzed by UPLC-MS/MS using a Waters Acquity UPLC system coupled to a Xevo TQD mass spectrometer. Compound concentrations were determined according to MS peak areas. Chromatographic separation was carried out using an Acquity BEH C18 column (1.7 μm, 2.1 × 100 mm). The mobile phase consisted of water containing 0.1% formic acid and acetonitrile containing 0.1% formic acid, delivered at a flow rate of 0.6 mL/min. The gradient program was as follows: 0.0–0.1 min, 100% water; 0.1–1.0 min, linear gradient to 100% acetonitrile; 1.0–2.0 min, 100% acetonitrile; 2.0–2.1 min, return to 100% water; and 2.1–2.5 min, 100% water for re-equilibration.

This analysis was performed at the Innopharma Drug Screening and Pharmacogenomics Platform, CiMUS Centre.

*CO23 treatment*

CO23 (HY-130012, MedChemExpress) was dissolved in dimethyl sulfoxide (DMSO, D2653-5x10ml, Merck) according to the manufacturer's instructions. For the *in vitro* experiments, a concentration-response analysis of CO23 was performed for LX-2. Based on these results, the final concentrations selected were 0.25, 0.5 and 1 nM for LX-2 during 24h.

*RNA-seq from PhHSCc*

Total RNA was extracted using the TRIzol–chloroform method following the manufacturer's protocol. RNA quality and quantity were assessed using a NanoDrop spectrophotometer. Only samples with A260/A280 ratios between 1.8 and 2.0 and no signs of degradation were used.

RNA samples were sent to BGI Genomics (Shenzhen, China), where RNA integrity was confirmed (RIN ≥ 8). Ribosomal RNA was depleted, and sequencing libraries were prepared and sequenced. Bioinformatic analysis, including quality control, alignment to the mouse reference genome, differential gene expression, and functional enrichment analysis, were performed using the EgoGenomics platform.

The RNA-seq data generated in this study have been deposited in the Gene Expression Omnibus (GEO) database under accession code GEO: GSE334408. Data reported in this paper will be shared by the lead contact upon request.

*Quantitative Proteomic by SWATH method.*

Protein extraction and digestion. Proteins from LX-2 cells were prepared by homogenization using a TissueLyser II (Qiagen) in cold RIPA buffer [containing 200 mmol/l Tris/HCl (pH 7.4), 130 mmol/l NaCl, 10%(v/v) glycerol, 0.1%(v/v) SDS, 1%(v/v) Triton X-100, 10 mmol/l MgCl2] with anti-proteases and anti-phosphatases (Sigma-Aldrich). The tissue lysates were centrifuged for 10 min at 14,000 g in a microfuge at 4ºC. The protein quantification was assessed for each sample using RC- DC kit (Bio-Rad) according to manufacturer's recommendations. 100 µg from each sample from each model was loaded on a 10% SDS-PAGE gel to whole-protein in gel concentration. Gel was staining and the band was exscinded and submitted to an in gel tryptic digestion. Peptides were extracted by

carrying out three 20-min incubations in 40 μl of 60% acetonitrile dissolved in 0.5% HCOOH, then pooled, concentrated (SpeedVac), and stored at −20 °C. Mass spectrometric analysis by SWATH (Sequential Window Acquisition of all Theoretical Mass Spectra). In order to construct the MS/MS spectral libraries each sample was equally pooled in control group, TGF-β1, TGF-β1 + Resmetirom 100 μM and siTHRα + TGF-β1 + Resmetirom 100 μM (n=4). All peptide solutions were analyzed by a shotgun data-dependent acquisition (DDA) approach by micro-LC- MS/MS, an analysis as previously described (2, 3). Therefore, ion density found in the DDA runs was used to create the necessaries windows in the SWATH method. Once the SWATH method is created the individual samples were run as described previously by our group [3, 4]. The targeted data extraction from the SWATH runs was performed by PeakView (version 2.2) using the SWATH Acquisition MicroApp (version 2.0) and the data was processed using the spectral library created previously from DDA runs. SWATH quantization was attempted for all proteins in the ion library that were identified by ProteinPilot with an FDR below 1%. PeakView computed an FDR and a score for each assigned peptide according to the chromatographic and spectra components; only peptides with an FDR below 1% 10 peptides and 7 transitions per peptide were used for protein quantization. The integrated peak areas were processed by MarkerView software (AB SCIEX) for data-independent method for relative quantitative analysis. To control for possible uneven sample loss across the different samples during the sample preparation process, we performed a MLR normalization (Most Likely Ratio). For each library, its set of differentially expressed proteins (p-value <0.05) with a 1.2-fold in- or decrease was selected. Data are available via ProteomeXchange with identifier PXD079822.

*Human anandamide assay kit*

Anandamide (AEA) levels were quantified using a competitive ELISA kit (MyBioSource, Cat. No. MBS7254670), following the manufacturer's instructions. Briefly, cells were detached using trypsin, collected by centrifugation, and resuspended in PBS. Cell suspensions (150.000 LX-2 cells: 100 μL; 24.000 phHSCs cells: 10 μL) were subjected to three freeze–thaw cycles to ensure lysis, followed by centrifugation at 3000 × g for 15 minutes to remove cellular debris. Supernatants were stored at −80 °C until analysis. For the assay, 100 μL of standards or supernatants were added to the antibody-coated wells, and 10 μL of Balance Solution was added to each sample well.

After thorough mixing, 50 μL of Conjugate was added to all wells except the blank control, and the plate was incubated at 37 °C for 1 hour. Wells were washed five times with 1× wash buffer, then 50 μL each of Substrate A and Substrate B were added sequentially. The plate was incubated for 15 minutes at 37 °C in the dark. The enzymatic reaction was terminated by adding 50 μL of Stop Solution to each well, and absorbance was measured at 450 nm using a microplate reader. AEA concentrations were determined by interpolation from a standard curve generated from known analyte concentrations.

*Real-time PCR*

RNA was extracted from cell samples using TRIzol reagent (Invitrogen) following the manufacturer' s instructions. A total of 100 ng of RNA was utilized for each reverse transcription (RT) reaction, and cDNA synthesis was performed using the SuperScript First-Strand Synthesis System (Invitrogen) with random primers. Negative control reactions, which contained all reagents except the RNA sample, were included to ensure the specificity of the PCR amplification. For real-time PCR, a fluorescent temperature cycler (Applied Biosystems) was used according to the manufacturer's guidelines, using TaqMan probes (Applied Biosystems, Life Technologies) or SYBR green Precision®PLUS reagent (PPLUS-LR, PrimerDesign). The TaqMan cycling conditions included an initial denaturation at 50° C for 10 minutes, followed by 45 cycles of denaturation at 95° C for 15 seconds and annealing/extension at 60° C for 1 minute. The SYBR green PCR cycling conditions included an initial denaturation at 95 °C for 2 min followed by 40 cycles at 95 °C for 10 s and 60 °C for 60 s. The oligonucleotide specific primers are shown in Table S2. All reactions were performed in duplicate using the QuantStudio 5 Real-Time PCR system (Applied Biosystems). Gene expression levels were normalized to HPRT1 or GAPDH for each sample according to the detection method used, and fold change values were calculated using the equation $2^{-\Delta\Delta Ct}$.

*Western blot analysis*

Total protein lysates from LX-2 and THLE-2 cells (6-9 μg, respectively) were subjected to SDS-PAGE, followed by electrotransfer onto polyvinylidene difluoride (PVDF) membranes (BioRad). The membranes were then incubated with the following primary antibodies: CC3 (1:1000, Rabbit, 9492S, Cell Signalling), CC7 (1:1000, Rabbit, 9661S,

Cell Signalling), GAPDH (1:1000, Mouse, ab9485, Abcam), and HSP90 (1:5000, Mouse, SC7947, Santa Cruz). For detection, horseradish peroxidase-conjugated secondary antibodies and chemiluminescence reagents (Amersham Biosciences) were employed. The membranes were exposed to radiographic film (Super RX Fuji Medical XRay Film, Fujifilm) and developed using developer and fixer solutions (AGFA) under appropriate dark room conditions. Protein expression was quantified through densitometric analysis using ImageJ software. Protein levels were normalized to GAPDH or HSP90 for each sample and expressed relative to the control group.

REFERENCES


1. da Silva Lima, N., et al., *GLP-1 and GIP agonism has no direct actions in human hepatocytes or hepatic stellate cells.* Cell Mol Life Sci, 2024. **81**(1): p. 468.
2. Novoa, E., et al., *Lack of PCK1 in hepatic stellate cells causes liver fibrosis by fueling tricarboxylic acid cycle and increasing glycolysis.* Cell Metab, 2026. **38**(4): p. 729-745 e9.
3. Fondevila, M.F., et al., *p63 controls metabolic activation of hepatic stellate cells and fibrosis via an HER2-ACC1 pathway.* Cell Rep Med, 2024. **5**(2): p. 101401.
4. Novoa, E., et al., *Mitochondrial antiviral signaling protein enhances MASLD progression through the ERK/TNFalpha/NFkappabeta pathway.* Hepatology, 2025. **81**(5): p. 1535-1552.
